# Comprehensive Biosafety Profile of Carbomer-Based Hydrogel Formulations Incorporating Phosphorus Derivatives

**DOI:** 10.3390/gels10070477

**Published:** 2024-07-18

**Authors:** Khaled Zakzak, Alexandra-Denisa Semenescu, Elena-Alina Moacă, Iasmina Predescu, George Drăghici, Lavinia Vlaia, Vicenţiu Vlaia, Florin Borcan, Cristina-Adriana Dehelean

**Affiliations:** 1 Department of Pharmaceutical Technology, Faculty of Pharmacy, “Victor Babes” University of Medicine and Pharmacy, 2nd Eftimie Murgu Square, 300041 Timisoara, Romania; khaled.zakzak@umft.ro (K.Z.); vlaia.lavinia@umft.ro (L.V.); 2 Department of Toxicology, Drug Industry, Management and Legislation, Faculty of Pharmacy, “Victor Babes” University of Medicine and Pharmacy Timisoara, 2nd Eftimie Murgu Square, 300041 Timisoara, Romania; alexandra.scurtu@umft.ro (A.-D.S.); iasmina-alexandra.predescu@student.umft.ro (I.P.); draghici.george-andrei@umft.ro (G.D.); cadehelean@umft.ro (C.-A.D.); 3 Research Center for Pharmaco-Toxicological Evaluation, “Victor Babes” University of Medicine and Pharmacy Timisoara, 2nd Eftimie Murgu Square, 300041 Timisoara, Romania; 4 Formulation and Technology of Drugs Research Center, “Victor Babes” University of Medicine and Pharmacy, 2nd Eftimie Murgu Square, 300041 Timisoara, Romania; vlaiav@umft.ro; 5 Department of Organic Chemistry, Faculty of Pharmacy, “Victor Babes” University of Medicine and Pharmacy, 2nd Eftimie Murgu Square, 300041 Timisoara, Romania; 6 Department of Analytical Chemistry, Faculty of Pharmacy, “Victor Babes” University of Medicine and Pharmacy, 2nd Eftimie Murgu Square, 300041 Timisoara, Romania; fborcan@umft.ro

**Keywords:** Carbopol 940, caffeine, in vitro assessment, RT-PCR, in vivo study, BALB/c nude mice, skin biophysical parameters, in ovo assay, HET-CAM

## Abstract

Determining the safety of a newly developed experimental product is a crucial condition for its medical use, especially for clinical trials. In this regard, four hydrogel-type formulations were manufactured, all of which were based on carbomer (Blank-CP940) and encapsulated with caffeine (CAF-CP940), phosphorus derivatives (phenyl phosphinic (CAF-S1-CP940) and 2-carboxyethyl phenyl phosphinic acids (CAF-S2-CP940)). The main aim of this research was to provide a comprehensive outline of the biosafety profile of the above-mentioned hydrogels. The complex in vitro screening (cell viability, cytotoxicity, morphological changes in response to exposure, and changes in nuclei morphology) on two types of healthy skin cell lines (HaCaT—human keratinocytes and JB6 Cl 41-5a—murine epidermal cells) exhibited a good biosafety profile when both cell lines were treated for 24 h with 150 μg/mL of each hydrogel. A comprehensive analysis of the hydrogel’s impact on the genetic profile of HaCaT cells sustains the in vitro experiments. The biosafety profile was completed with the in vivo and in ovo assays. The outcome revealed that the developed hydrogels exerted good biocompatibility after topical application on BALB/c nude mice’s skin. It also revealed a lack of toxicity after exposure to the hen’s chicken embryo. Further investigations are needed, regarding the in vitro and in vivo therapeutic efficacy and safety for long-term use and potential clinical translatability.

## 1. Introduction

The development of new effective and safe biopharmaceutical products is a long process that begins with the identification of the therapeutic active substance (chemical or natural) and ends when the product is approved and marketed. Determining the safety and efficacy of an experimental biopharmaceutical product is a mandatory condition in preclinical development for determining its feasibility for medical use [1,2]. During preclinical development, continuous supervision is also mandatory as it includes the activities carried out from the discovery of the biopharmaceutical product in the laboratory and the initiation of clinical trials on humans, to ensure their safety and wellness in the study [3]. Since 1990, the International Council for Harmonization (ICH) has harmonized the regulations governing the development and marketing of medicines [4], and its priority was developing guidelines on the safety, quality, and efficacy of medicines. The United Nations Educational, Scientific and Cultural Organization (UNESCO) and the World Health Organization (WHO) signed an agreement, after which the Council for International Organizations of Medical Sciences (CIOMS) was born [5]. After about four decades since was set up, the CIOMS has established working groups to focus on drug pharmacovigilance, because any drug-related problem based on research or activities about detection, assessment, and prevention of adverse effects of a drug must be reported [6]. Therefore, it is extremely important to assess first the biosafety of newly developed biopharmaceutical products to determine further feasibility of their medical use. 

Nowadays, toxicological research is mandatory in the biological assessment of potential drugs, biopharmaceutical products, and medical devices. The reason for this is that possible adverse effects of the test compound can be identified and prevented through in vitro experiments and in vivo toxicity testing [7,8]. Concerning the in vivo screening, preclinical toxicology studies refer both to its safety assay and to the identification of the nature and severity of potential harmful effects of pharmacologically active compounds, which are signaled on the body of an experimental model [9].

Hydrogels are three-dimensional (3D) polymeric networks that have been extensively investigated especially due to their tunable physicochemical properties, biocompatibility, semi-solid structure, and their ability to encapsulate various compounds, drugs, materials, cells, etc. This has led to their widespread use in diverse applications including biomedicine. There are two types of hydrogels: natural and synthetic; the difference between them is related to the mechanical properties, which are relatively higher in the case of synthetic hydrogels [10]. 

Carbomer, a synthetic polymer of cross-linked polyacrylic acid, is widely used for its versatility and effectiveness as a gelling agent in various applications, particularly in pharmaceuticals, cosmetics, and food products [11,12,13,14]. As a gelling agent, offers numerous advantages, such as the ability to create high-viscosity gels at relatively low concentrations, bio-adhesive properties, thermal stability, low potential for skin irritation, organoleptic properties [15], and compatibility with a wide range of active ingredients [16,17,18,19,20,21,22], making it the first choice for the development of various formulations [13,23,24,25]. The high viscosity of carbomer-based hydrogels also contributes to prolonged drug retention, enhancing the bioavailability of medications, especially in ocular applications [26,27]. Studies have shown that carbomers, such as Carbopol, can act as stabilizers, suspending agents, and controlled-release agents in pharmaceutical products, demonstrating their versatility in different dosage forms [28,29,30]. 

Caffeine plays a multifaceted role in pharmaceutical technology, encompassing aspects of formulation, delivery, and adsorption, being widely explored due to its diverse applications and properties. According to this, several studies have highlighted the benefits of incorporating caffeine into hydrogel formulations. For instance, loading caffeine as a model drug into chitin-glucan complex hydrogels led to an improvement in the mechanical and rheological properties of the hydrogels [31]. In addition, caffeine as an ingredient has demonstrated its role in drug-controlled release capabilities from different types of hydrogels [32,33], as well as its ability to modulate drug release profiles within hydrogel matrices [34,35]. Moreover, caffeine’s versatility was also demonstrated in pharmaceutical and cosmetic applications. In a research study, Luo and Lane [36] assessed the topical and transdermal delivery of caffeine, showing that when administered topically, can serve as a model hydrophilic compound for assessing dermal risk in drug delivery systems. Narayanan and co-workers [37] evaluated a skin-invigorating caffeine facemask, emphasizing the multi-purpose utility of caffeine-containing hydrogels in cosmetic applications. Dong and co-workers [38] evaluated the adsorption behavior of catechins and caffeine onto polyvinylpolypyrrolidone, underscoring the importance of purification processes in pharmaceutical applications. Purifying compounds before formulation is essential to ensure drug efficacy and safety. As regards the dermatology field, Rosado and co-workers [39] conducted in vivo studies confirming that formulations combining caffeine with sunscreen filters resulted in higher SPF values compared to caffeine-free samples, indicating the potential of caffeine as a sunscreen adjuvant. Additionally, Alsamad [40] focused on the directional assessment of skin barrier function in vivo using caffeine as a marker, highlighting the utility of caffeine in studying skin physiology and barrier integrity. Overall, caffeine, a widely consumed psychoactive substance, also presents challenges in biomedical applications. For example, caffeine as a non-selective adenosine antagonist interacts with these receptors and influences various physiological and psychological processes related to anxiety and stress [41]. Another challenge of caffeine in biomedical applications is associated with the complexity of its interactions with liver cells [42]. However, understanding the complex mechanisms of caffeine can provide insights into its potential therapeutic applications and challenges in managing anxiety-related conditions as well as the importance of dose-dependent effects interactions with liver cells. 

The derivatives of hypophosphorous acid (H_2_P(O)(OH)) were introduced in 1855 by August Wilhelm Hofmann [43], and among them, the phosphinic acid compounds are also counted. It has been stated that this organophosphorus derivatives class has a plethora of applications in biochemistry and biology, including in medicine [44,45,46,47]. In addition, to obtain polymeric-related structures characterized by different dimensionalities [48], the phenyl phosphinic acids were used to obtain hybrid materials complexes (organic/inorganic) [49]. 2-carboxyethyl phenyl phosphinic acid—prepared for the first time by Birum and Jansen [50] from dichloro (phenyl)phosphine and acrylic acid, was used as a reagent to obtain one-, two- or three-dimensional porous polymeric structures (metal clusters coordinated with organic ligands) [51] or phosphorus-based flame retardants [52]. Phosphinic acids have gained increasing attention for their applications in industrial, agricultural, medicinal, and synthetic intermediate sectors [53]. Additionally, α-aminophosphonic acid derivatives, mimicking tetrahedral intermediates of hydrolyzed esters, amides, and peptides, have found applications as antibiotics, herbicides, antitumor agents, and enzyme inhibitors in agriculture and medicine [54]. The potential of aminophosphonic acids in medicinal chemistry remains remarkable, highlighting their versatility and importance in drug development [55]. The phosphorus substituents have been found to modulate crucial biological functions, with molecular alterations significantly affecting biological activity [56]. Furthermore, the presence of phosphorus substituents has been demonstrated to modulate the biological activities of pesticides, antiviral agents, and antimicrobial compounds, highlighting the crucial role of phosphorus derivatives in pharmaceutical research [57]. In addition, the synthesis of β-aminophosphonates and β-phosphinates has contributed to the preparation of phosphorus analog peptides, emphasizing the utility of these derivatives in constructing bioactive compounds [58]. A significant challenge of these compounds in biomedical applications is the tendency of acidic phosphorus-containing functional groups, such as phosphates, phosphonates, and phosphinates, to exist as charged species at physiological pH. This characteristic can lead to issues like low cell permeability and poor oral bioavailability [59]. Despite these challenges, phosphorus-based nanomaterials have gained attention for biomedical use (e.g., monomers like red phosphorus, black phosphorus, violet phosphorus, and blue phosphorus) [60]. Moreover, it was stated that phosphorus compounds substituted by 1,2,4-triazine moieties have been studied for their medicinal properties, thus proving that through diverse chemical modifications made to phosphorus structures, the biological effects of these compounds can be tailored [61]. Overall, the versatilities of phosphorus derivatives in medicine were also demonstrated through the synthesis of hydroxyl and amino-phosphonates and –bisphosphonates, which has led to the development of compounds with distinct features suitable for various therapeutic applications [62]. 

The incorporation of phosphorus derivatives in carbomer-based hydrogel formulations introduces a unique dimension to their physicochemical properties, potentially influencing their toxicity profiles. Although phosphorus compounds have been associated with various toxicological concerns, from low to high toxicity, including acute toxicity, organ damage, and environmental hazards [63], phosphorus derivatives are essential in biomedical applications due to their unique properties and diverse functionalities. Therefore, it is imperative to thoroughly investigate the impact of these derivatives on the overall safety of hydrogel formulations. 

In the context of the above, the present study highlights the importance of assessing the potential toxicity of carbomer-based hydrogel formulations containing a mixture of caffeine and phosphorus derivatives on relevant cell lines to ensure their safety for further biomedical applications. We have prepared and investigated synthetic hydrogels based on caffeine (0.5 wt%) and phosphorus compounds. In addition, we have evaluated the hydrogel’s effects on primary healthy human/murine cells (in vitro) to ensure their safe use and assessed the hydrogel’s in vivo biocompatibility using an experimental model (BALB/c nude mice) in which we examined the variation of mice’s skin parameters following topical application. Moreover, it was performed via an embryotoxicity test, using an in ovo HET-CAM assay (hen’s eggs chorioallantoic membrane test) to understand the possible consequences of the potential use of the prepared hydrogel as well as special aspects of systemic toxicity. 

To our knowledge, the present study represents the first study dealing with this hydrogel-type formulation incorporating phosphorus compounds (phenyl phosphinic and 2-carboxyethyl phenyl phosphinic acids). Since the carbomer-based hydrogel formulations that were obtained in the current study represent an element of originality (no such formulations have been prepared and no biological studies were carried out, as far as we know), testing the hydrogel’s in vivo effects became mandatorily. Therefore, the newness of this study relies on performing a comprehensive toxicological profile (in vitro experiments, in vivo biocompatibility tests, and an in ovo toxicity assessment). After this, we will be able to establish the biosafety profile of the as-prepared carbomer-based hydrogel formulations, which contain caffeine and phosphorus derivatives. By conducting comprehensive toxicological screening, this research aims to provide a holistic understanding of the biosafety profiles of these novel formulations, guiding future research directions and regulatory decisions.

## 2. Results and Discussion

### 2.1. Selection of Gelling Agent for the Preparation of the Hydrogels Containing a Caffeine-Phosphorus Derivative Mixture

A gelling agent is required to convert aqueous drug solutions into hydrogels, which are preferred for topical application. The selection criteria of the suitable gelling agent for aqueous solutions of caffeine-phosphorus derivatives combinations were the polymer’s compatibility with active ingredients and the pH of the solution. Considering that the aqueous solutions of caffeine-phosphorus derivatives combinations were acidic (pH values between 3.32 and 3.71), three hydrophilic polymers namely chitosan, colloidal silicon dioxide, and carbomer 940 were chosen for screening, based on their known stability at low pH values. It was observed that chitosan was ineffective as a gelling agent because of its partial dissolution in the acidic aqueous solutions. This inefficiency could be attributed to the decrease of its water solubility, which thus reduces its thickening ability in the presence of inorganic phosphorus derivatives [64] and in aqueous solutions as phosphorus anion species. Similarly, the expected thixotropic thickening effect of colloidal silicon dioxide when used in appropriate concentrations (2–10 wt%) in aqueous systems with pH values between 0 and 7.5 [64] could not be achieved after its addition in aqueous solutions of caffeine-phosphorus acid derivatives. The unsuccessful attempt to thicken our aqueous solutions was most probably due to the interference of phosphorus derivatives in the colloidal silicon dioxide gelation process. Unlike these two tested polymers, carbomer 940 (Carbopol 940—CP940) in 1 wt% concentration gelled the aqueous solutions, acting as an efficient thickening agent simply by its dispersion and hydration into the liquid, without the need to neutralize it by adding a base (usually sodium hydroxide or triethanolamine). Based on these observations, carbomer 940 in 1 wt% concentration was selected as a gelling agent to obtain several hydrogels containing caffeine-phosphorus derivatives mixtures. 

With regard to the phosphorus derivatives used, those are phenyl phosphinic acid (S1)—a natural product containing carbon-phosphorus bonds, and 2-carboxyethyl phenyl phosphinic acid (S2)—prepared from dichloro(phenyl)phosphine and acrylic acid by our group of research [65]. These two organophosphorus compounds were selected based on the many applications both in biochemistry and biology, as well as in medicine [44,45]. Further, the hydrogels containing caffeine (CAF) and phosphorus derivatives will be referred to as follows: CAF-S1-CP940 and CAF-S2-CP940. 

### 2.2. Characterization of Carbomer-Based Hydrogel Formulations

#### 2.2.1. Determination of Macroscopic Properties and pH

**Figure 1:**
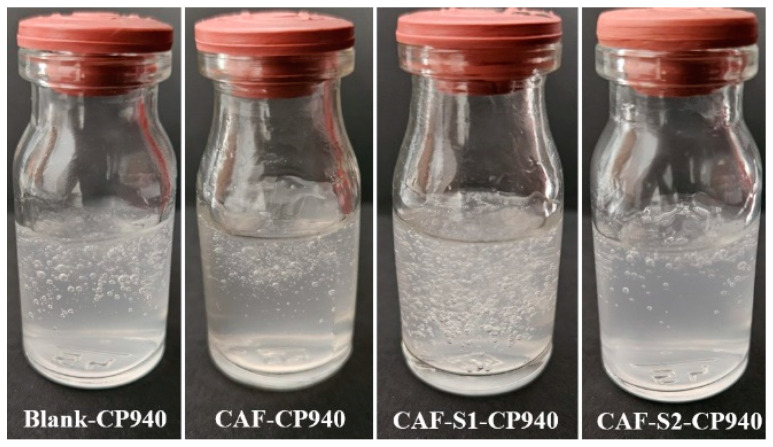
Macroscopic appearance of experimental carbomer-based hydrogels.

The visual assessment showed that before phosphorus derivatives (S1 and S2) incorporation, Blank-CP940 (control formulation) and CAF-CP940 (the carbomer-based hydrogel containing 0.5 wt% caffeine (CAF)) formulations were slightly opalescent, colorless, odorless, and in soft gel state at room temperature (Figure 1). The hydrogel’s opalescence is due to carbomer dispersion in the colloidal state. After incorporating caffeine (CAF) with phosphorus derivatives S1 and S2, the obtained hydrogels became transparent and maintained their homogenous aspect and consistency, confirming the complete dissolution of active ingredients and acrylic polymer.

The pH value of 1 wt% carbomer 940-based hydrogel (Blank-CP940) was 3.96 ± 0.029 (Table 1), which falls into the range specified in the literature [64,66] and is due to the acidic character of the polyelectrolyte (carbomer 940 is a synthetic polymer, obtained by crosslinking acrylic acid with allyl ethers of pentaerythritol) [67]. The carbomer-based hydrogel containing 0.5 wt% caffeine (CAF-CP940 formulation) showed a pH value about one unit higher than the pH of the control hydrogel (Table 1) as caffeine is a weak base and more probably partially neutralizes the polyelectrolyte based on acid-base interaction, mechanism demonstrated in literature for other weak basic drugs [68,69,70]. In contrast, the pH values determined for the carbomer-based hydrogel formulations containing a combination of caffeine with phosphorus derivatives S1 and S2 (CAF-S1-CP940, and CAF-S2-CP940, respectively) ranged from 3.32 ± 0.048 to 3.71 ± 0.052 (Table 1), being lower than that of Blank-CP940 and of CAF-CP940 formulations. This increase in hydrogel acidity can be attributed to the strong acidic character of phosphorus compounds (S1—phenyl phosphinic acid and S2—2-carboxyethyl phenyl phosphinic acid) [46].

Among the hydrogels that were tested, only the CAF-CP940 formulation obtained a pH value acceptable for its application on the skin (4.5—8) [71] consequently, indicating a safe topical preparation. However, recently published studies on topical acidic creams containing glycolic acid suggested that acidification of the skin surface could be induced after their application, without signs of irritation or barrier impairment [72]. Therefore, in the case of acidic hydrogel formulations CAF-S1-CP940 and CAF-S2-CP940, the investigation of their effect on skin health would be necessary before considering their acidity as an inconvenience.

#### 2.2.2. Rheological Measurements

**Figure 2:**
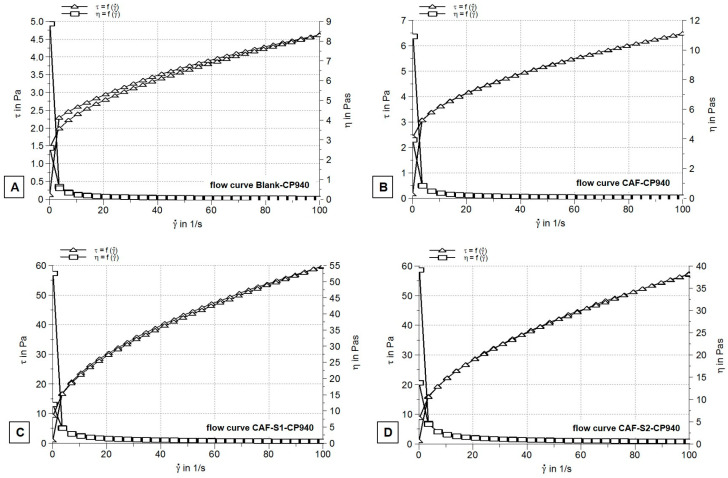
Rheograms and viscosity curves for experimental un-neutralized Carbopol 940-based hydrogels: (**A**)—Blank-CP940; (**B**)—CAF-CP940; (**C**)—CAF-S1-CP940, and (**D**)—CAF-S2-CP940.

Rheograms recorded after performing steady-state flow test for all un-neutralized 1 wt% Carbopol 940-based hydrogels (Blank-CP940) (Figure 2A) showed non-Newtonian time-independent flow with plastic profile and negligible thixotropy. 

The specific features of this type of system are the following: flow behavior depends on shear rate and not on the period of shear; a critical stress value (yield stress) needs to be attained to initiate the flow (after that, the flow continues to increase); and, finally, the viscosity decreases by increasing shear stress (shear thinning effect) until a plateau is reached. The plastic behavior of experimental hydrogels indicates that under shearing conditions of the test, their structure was not broken down or disrupted, it reformed very quickly [73,74]. 

The decrease in viscosity observed from the recorded viscosity curves (Figure 2A,B) of the studied hydrogels and the little to no thixotropy (showed by their flow curves (Figure 2C,D)) are more likely due to the network structure of the un-neutralized systems formed mainly by hydrogen bonding, which are easily broken down [73,74].

Furthermore, it can be observed that, in all cases, the shear thinning effect occurred at low shear rates. The apparent viscosity value of un-neutralized Carbopol 940 hydrogel (Blank-CP940) was 0.046 ± 0.16 Pa.s, very close to that previously indicated in the literature [73]. A slightly higher apparent viscosity value, though still within the same order of magnitude (0.065 ± 0.34), was 0.5 wt% for the caffeine-loaded hydrogel (CAF-CP940) (Figure 2B), most probably because of its loose network structure formed by the partial neutralization with caffeine. On the contrary, the viscosity values of the formulations containing a combination of caffeine with phosphorus derivatives S1 and S2 were about 10-fold higher than those of Blank-CP940 and caffeine-loaded hydrogel formulations (CAF-CP940) (Table 1). As an explanation for these results, one can suggest a different thickening mechanism of carbomer 940 in the presence of phosphorus derivatives. In addition, no significant differences were observed between the viscosity values of the phosphorus hydrogel formulations CAF-S1-CP940 (Figure 2C), and CAF-S2-CP940 (Figure 2D).

Our findings are in agreement with the outcomes of the other studies, indicating that the obtained hydrogels possess pseudoplastic rheology being non-Newtonian systems [75,76,77]. This type of system and rheological property indicate that the gel’s viscosity decreases in high shear, facilitating its topical application.

The rheological behavior of carbomer-based hydrogels is influenced by multiple factors, including pH levels. Studies have shown that the pH of carbomer hydrogels affects their rheological, textural, and release properties. As the pH increases, the carbomer molecules’ globules unfold into a bulk network, resulting in increased system viscosity [78]. Furthermore, the introduction of liposomes containing compounds like caffeic acid can alter the rheological properties of carbomer hydrogels, influencing parameters such as viscosity, consistency, and thixotropy [79]. The incorporation of compounds containing phosphate groups, like phospholipids, can significantly modify the rheological properties of carbomer hydrogels [79]. These changes have an impact on the structural integrity, flow behavior, and overall performance of the hydrogels, making them more adaptable and tailored to specific applications. 

#### 2.2.3. Spreadability Test

The spreadability was evaluated in the current study, to determine the tegument ease of application of semisolid formulations since is a key characteristic for patient acceptability, being also a rheological property related to consistency [80]. In the present study, the high spreadability values obtained (Table 1) suggest that the hydrogel formulations are easily spreadable systems at 25 °C. In addition, the Blank-CP940 and CAF-CP940 formulations obtained significantly higher spreadability values (4654.27 ± 3.091 and 5671.63 ± 3.898, respectively), indicating a much lower consistency than that of carbomer-based hydrogels containing phosphorus derivatives. The spreadability values exhibited by Blank-CP940 formulation (un-neutralized Carbopol 940 hydrogel) and by CAF-loaded hydrogel formulations (Carbopol 940 partially neutralized by caffeine) were similar to those reported by Coneac and co-workers [81], who investigated several fluconazole microemulsion-loaded hydrogels based on Carbopol EDT 2020 (a cross-linked polyacrylic acid copolymer), also in un-neutralized form.

The rheological characterization of carbomer-based hydrogels is crucial for understanding their performance in specific applications. It was stated that carbomer gels exhibit unique elastoviscoplastic properties and low toxicity, which makes them valuable as thickening, suspending, dispersing, and stabilizing agents [82]. Moreover, the rheological properties of carbomer-based hydrogels can also influence their bio-stability and efficacy in wound healing applications. By adjusting the elasticity and viscosity properties of carbomer-based hydrogels, additives can enhance their plasticity, adhesiveness, and wound contact, thereby improving their therapeutic potential [83]. The significance of rheological parameters in controlling drug release profiles and efficacy was also highlighted, thus emphasizing the complex relationship between rheological properties, formulation components, and functional outcomes in carbomer-based hydrogels [84].

#### 2.2.4. Scanning Electron Microscopy Analysis

**Figure 3:**
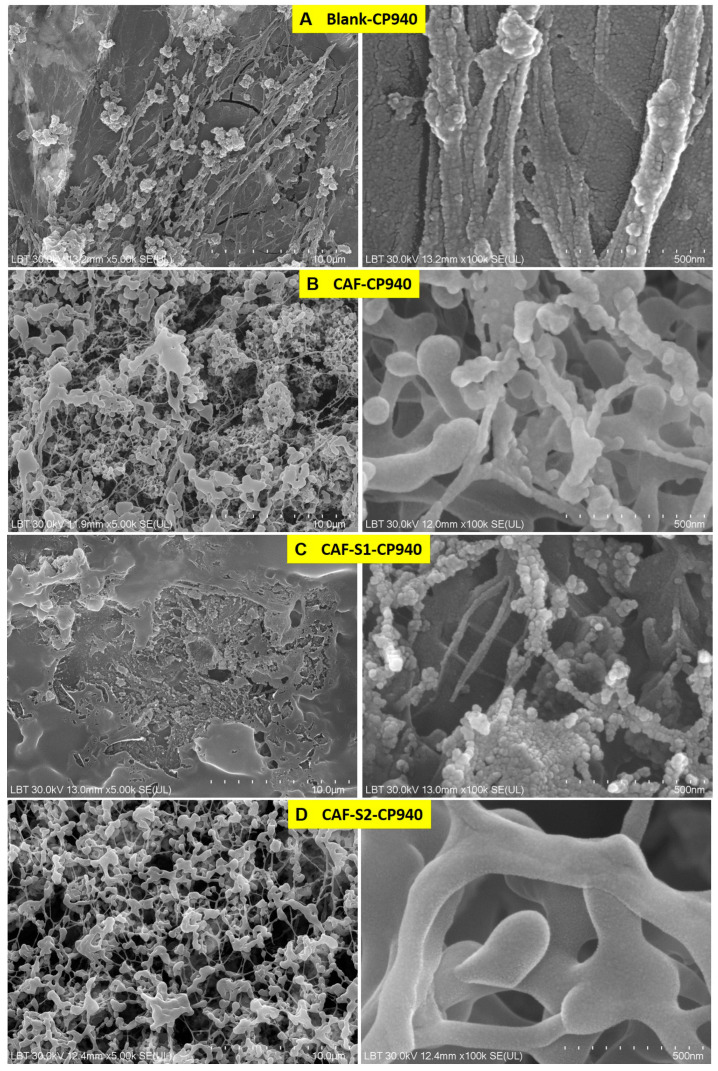
SEM images of (**A**)—Blank-CP940; (**B**)—CAF-CP940; (**C**)—CAF-S1-CP940, and (**D**)—CAF-S2-CP940.

Figure 3 shows representative images of carbomer-based hydrogel formulations containing caffeine and phosphorus derivatives at different orders of magnitude—one image as a general overview (×5.0 k) corresponding to the surface (left micrographs) of each hydrogel obtained and another image at higher magnification (×100 k) corresponding to internal morphologies of the hydrogels formulations (right micrographs).

As one can observe from Figure 3, all the carbomer-based hydrogel formulations showed more or less porous structure, denoting variate-swelling degrees of the developed hydrogel formulations. As regards the Blank-CP940 of 1% carbomer (Figure 3A), the SEM images showed that carbomer-based hydrogel possesses small mesh pores. In addition, slight network fissures could also be visible. This phenomenon may be due to the drying process of the carbomer-based hydrogel, which causes the fragmentation of the polymeric network. Moreover, at the magnification of ×5.00 k (left image), one can observe that the Blank-CP940 surface becomes rough in the same place where the collapse occurred [85]. Therefore, the Blank-CP940 revealed the presence of a rough, fissured, and porous structure with small meshes (Figure 3A).

On the contrary, the CAF-CP940 hydrogel, which was based on 1% carbomer with 5% caffeine content (Figure 3B), showed a high degree of porosity when compared with Blank-CP940, with various dimensions of pores that are interconnected between them [86]. Such a porous structure ensures sufficient space for both loading large amounts of compounds/drugs and for the compounds/drugs movement throughout the gel matrix, thus leading to an enhanced release rate [86]. The benefits of incorporating caffeine into hydrogel formulations were highlighted due to its ability to improve the hydrogel’s mechanical and rheological properties [31], its demonstrated controlled release capabilities from different types of hydrogels [34], as well as the impact of its concentration on the release kinetics. This emphasizes caffeine’s role as a key factor in determining drug-release behavior from hydrogel networks [87].

As regards the CAF-S1-CP940 (Figure 3C), the carbomer-based hydrogel formulation containing caffeine and phenyl phosphinic acid (S2), the SEM micrographs revealed a smooth structure at ×5.00 k magnification (left image) with a high degree of irregularity and heterogeneities. The heterogeneous network exhibits visible gel-fused spheres [88]. The image at the high magnification (right image) also consists of fused spherical regions and fiber diameter, which reveals deformation in their structures. The pores in this hydrogel formulation may enlarge upon swelling in water until they distort and polymer deformation occurs [89]. 

The CAF-S2-CP940 hydrogel formulation (Figure 3D), revealed a porous polymeric structure, with large spaces for water intake. The porous polymeric network leads to a high swelling degree; therefore, the pores carrying water could penetrate the polymeric network through some channels, thus enhancing the interactions between the water molecules with functional groups on the polymeric chain [90]. 

### 2.3. In Vitro Toxicological Screening of Carbomer-Based Hydrogel Formulations

#### 2.3.1. Cell Viability Assay

**Figure 4:**
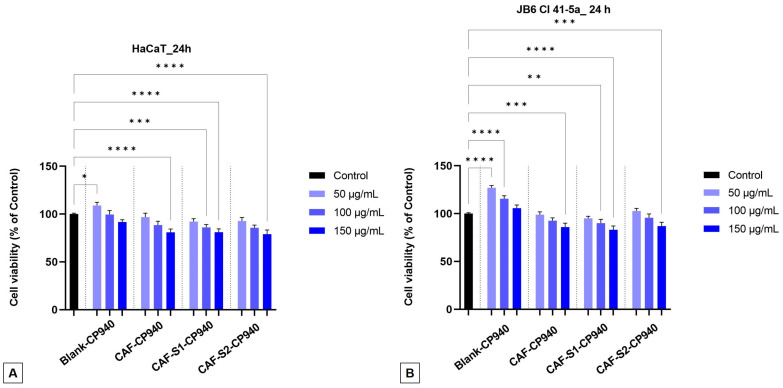
The cellular viability graphical representation at 24 h after treating (**A**)—HaCaT and (**B**)—JB6 Cl 41-5a cells with carbomer-based hydrogel formulations. The experiments were performed in triplicate and the mean value ± SD was calculated from three individual experiments. The one-way ANOVA test followed by Dunnet’s multiple comparison post hoc test was employed to analyze the statistical differences that occurred (* *p* ˂ 0.05; ** *p* < 0.01; *** *p* ˂ 0.001; **** *p* < 0.0001).

The impact on the cellular viability was assessed using the MTT analysis after the HaCaT (Figure 4A) and JB6 Cl 41-5a (Figure 4B) cells were treated with carbomer-based hydrogels: Blank-CP940, CAF-CP940, and two samples containing a mixture of caffeine and phosphorus derivatives (CAF-S1-CP940 and CAF-S2-CP940), respectively, for 24 h. After performing the MTT test on the two healthy skin cell lines, we noticed that the samples’ cell viability did not decrease by a large percentage. For the HaCaT cells (Figure 4A), the sample CAF-S1-CP940, which contained caffeine and phenyl phosphinic acid, decreased its cell viability by ≈81% when tested at the highest concentration (150 μg/mL). Meanwhile, the sample CAF-S2-CP940 (which contained caffeine and 2-carboxyethyl phenyl phosphinic acid) recorded a cell viability value of 78% when tested at the same concentration.

Regarding the JB6 Cl 41-5a cells (Figure 4B), we observed viability percentages of 83% and, respectively, 86% at the concentration of 150 µg/mL for the carbomer-based hydrogels containing caffeine and phosphorus derivatives (CAF-S1-CP940 and CAF-S2-CP940). As regards the effect of CAF-S2-CP940 hydrogel (sample containing a combination of caffeine and 2-carboxyethyl phenyl phosphinic acid) on JB6 Cl 41-5a cells, a slight cell proliferation could also be observed (102%) when the concentrations of 50 and 100 μg/mL were tested. Similar viabilities were recorded for Blank-CP940 and CAF-CP940 hydrogels on both cell lines, with a smaller decrease than those of hydrogels containing caffeine and phosphorus derivatives.

The impact of hydrogels on human healthy cell viability represents a significant area of research since healthy cells are influenced by various factors such as polymer density, gel composition, mechanical properties, porosity, and gel-cell interactions. Several studies have explored how different types of hydrogels affect cell survival and proliferation. For instance, Zustiak and co-workers [91] found that the total polymer density within hydrogels significantly influences cell viability, with higher densities leading to decreased viability likely due to changes in the hydrogel’s mechanical properties. Additionally, Gentilini and his co-workers [92] demonstrated that the pH and rheological properties of hydrogels could affect cell viability, showing that an acidic pH and stiffer network structures lead to lower cell viability. Hernandez and co-workers [93] showed that non-Newtonian polymer-nanoparticle hydrogels could enhance cell viability post-injection compared to traditional cell suspension methods. Moreover, it was stated that the interaction between cells and gelatin microparticles within hydrogels and the gelation mechanism employed could positively influence long-term cell viability [94]. 

Another important factor that could contribute to the influence of healthy cell viability is the polymer nature. It was stated that natural polymers like collagen, gelatin, and matrigel have been found to enhance cell viability and support specific cellular functions within hydrogel environments [95]. Although Carbopol is a synthetic polymer, several studies indicate the potential benefits of Carbopol-based constructs in promoting cell health (enhanced cell viability, biocompatibility, and cell infiltration observed with cross-linked hydrogels) [96], as well as its importance in tissue engineering applications, due to its favorable rheological behavior and cell-friendly environment [97]. Hayati and co-workers [98] evaluated the cytotoxicity of Carbopol 940 hydrogel on fibroblast cells to establish its efficiency in topical applications and found no significant differences between control and hydrogel, concluding that carbomer 940 is not toxic on fibroblasts. Moreover, Mair and co-workers [99] suggested a potential positive impact of Carbopol on immune responses proving that the synthetic polymer could enhance cellular immunity by promoting early interferon-gamma (IFN-γ) production and influencing T-cell differentiation towards effector phenotypes. On the other hand, a research study that evaluated the cytotoxicity of titanium dioxide- and gold-based Carbopol nanogels on normal human diploid cell lines provided insights into the potential adverse effects on cell viability [100].

Overall, one can conclude that the effects of Carbopol on healthy human cells and its viability for medical use are multifaceted, with studies indicating both potential benefits in enhancing immune responses and cell health, as well as concerns regarding cytotoxicity in certain formulations. According to this, it is crucial to understand the specific context of Carbopol use, its formulation characteristics, and cellular responses and assess its overall impact on human cells and its viability for medical use. 

#### 2.3.2. Cellular Morphology, Confluence, and Cell Number Evaluation

**Figure 5:**
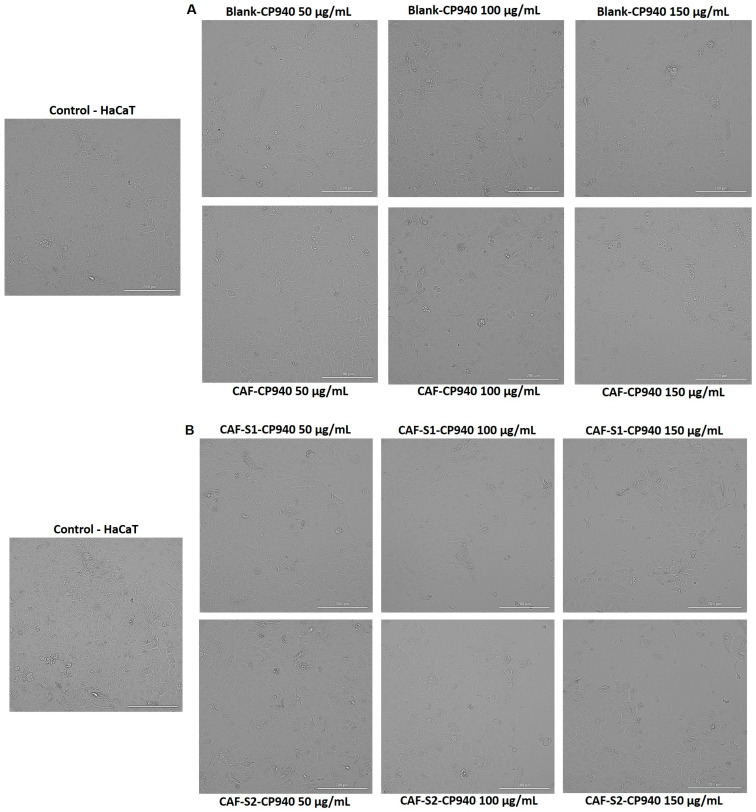
Morphological aspects at 24 h post-exposure of healthy human keratinocyte cell line (HaCaT) to (**A**)—Blank-CP940 and CAF-CP940 hydrogels (50, 100, and 150 μg/mL); and to (**B**)—CAF-S1-CP940 and CAF-S2-CP940 hydrogels (50, 100, and 150 μg/mL). Scale bars represent 200 µm.

**Figure 6:**
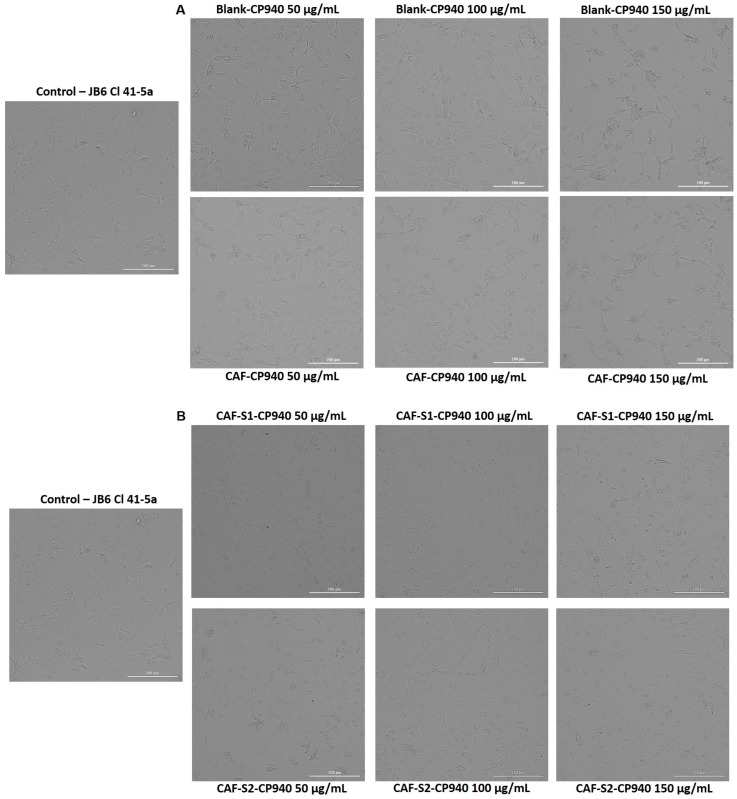
Morphological aspects of neonatal BALB/c epidermal cell line (JB6 Cl 41-5a) at 24 h post-exposure to (**A**)—Blank-CP940 and CAF-CP940 hydrogels (50, 100, and 150 μg/mL); and to (**B**)—CAF-S1-CP940 and CAF-S2-CP940 hydrogels (50, 100, and 150 μg/mL). Scale bars represent 200 µm.

To complete the cytotoxic profile of the carbomer-based hydrogel formulations, the impact of the samples on HaCaT (Figure 5) and JB6 Cl 41-5a (Figure 6) cells’ morphology was examined. As presented in Figure 5 (A—Blank-CP940 and CAF-CP940 hydrogels; and B—CAF-S1-CP940 and CAF-S2-CP940 hydrogels), in Figure 6 (A—Blank-CP940 and CAF-CP940 hydrogels (50, 100, and 150 μg/mL), and with the hydrogels (B—CAF-S1-CP940 and CAF-S2-CP940 hydrogels), the results of the analysis of the cell morphology claimed that on both cell lines, no significant morphological damage was noticed when compared with the control cells, following 24 h of treatment of all of the samples. However, as the concentration of the carbomer-based hydrogel formulations increased, changes were observed regarding the cell’s shape, meaning that the cells became rounder and detached from the plate and slight debris was observed.

Compared with our previously reported study, when the two phosphorus derivatives were evaluated (S1—phenyl phosphinic acid and S2—2-carboxyethyl phenyl phosphinic acid), one can observe that following the treatment of HaCaT cells with both carbomer-based hydrogel formulations containing caffeine and phosphorus derivatives, the morphology of the cells depicted in Figure 5B did not show significant alterations when compared with the control cells [65].

**Figure 7:**
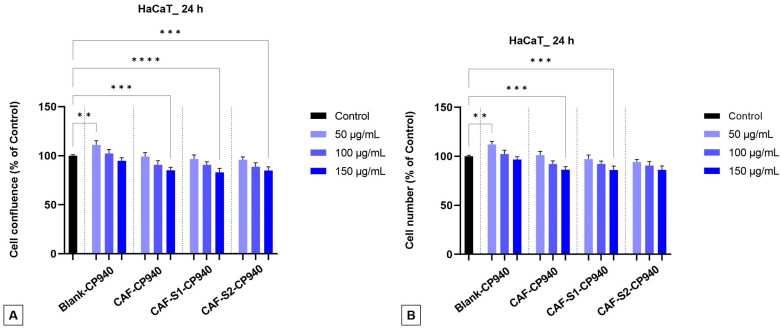
The cell confluence (**A**) and cell number (**B**) graphical representation at 24 h after treating HaCaT cells with carbomer-based hydrogel formulations. The experiments were performed in triplicate and the mean value ± SD was calculated from three individual experiments. The one-way ANOVA test followed by Dunnet’s multiple comparison post hoc test was employed to analyze the statistical differences that occurred (** *p* < 0.01; *** *p* < 0.001; **** *p* < 0.0001).

**Figure 8:**
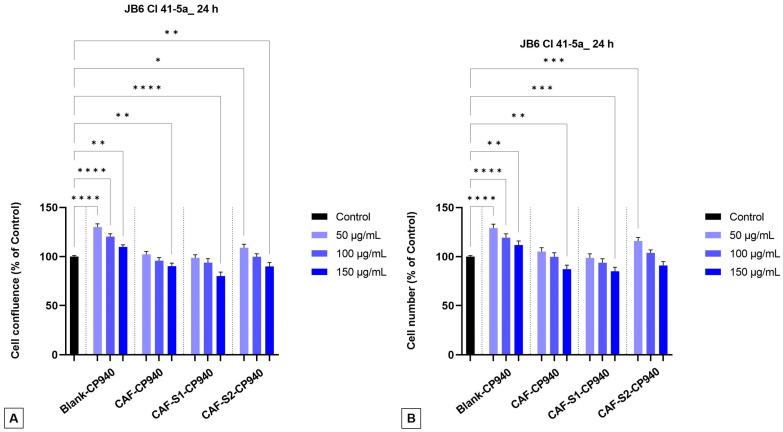
The cell confluence (**A**) and cell number (**B**) graphical representation at 24 h after treating JB6 Cl 41-5a cells with carbomer-based hydrogel formulations. The experiments were performed in triplicate and the mean value ± SD was calculated from three individual experiments. The one-way ANOVA test followed by Dunnet’s multiple comparison post hoc test was employed to analyze the statistical differences that occurred (* *p* ˂ 0.05; ** *p* < 0.01; *** *p* < 0.001; **** *p* < 0.0001).

Further, the impact of carbomer-based hydrogel formulations, containing caffeine and phosphorus derivatives, as well as Blank-CP940, and CAF-CP940 was investigated on both the confluence, as well as the number of cells. One can observe that with the increase of sample concentrations on both cell lines—HaCaT and JB6 Cl 41-5a, a decrease in confluency (Figure 7A and Figure 8A) as well as in cell number (Figure 7B and Figure 8B) occurs. The lowest percentages (≈85%) were exhibited for the carbomer-based hydrogel containing caffeine and phenylphosphinic acid (CAF-S1-CP940), on the HaCaT cell line (Figure 7A,B).

#### 2.3.3. Cytotoxicity Assay

The lactate dehydrogenase method (LDH) was employed to determine the cytotoxicity of the carbomer-based hydrogel formulations. The cytotoxic effect (or more precisely, the impact of the hydrogels on the integrity of the cell membrane) was evaluated by quantifying the release of LDH. 

**Figure 9:**
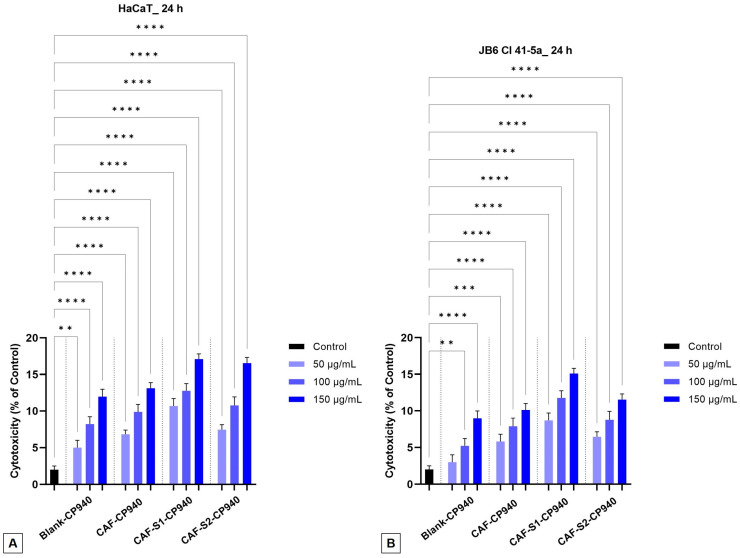
Cytotoxicity percentages of healthy human keratinocytes HaCaT (**A**) and neonatal BALB/c epidermal cells JB6 Cl 41-5a (**B**), after treatment with carbomer-based hydrogel formulations (50, 100, and 150 μg/mL). At 24 h post-stimulation, the LDH assay was performed. The experiments were performed in triplicate and the mean cytotoxic value ± SD was calculated from three individual experiments. The one-way ANOVA test followed by Dunnet’s multiple comparison post hoc test was employed to analyze the statistical differences that occurred between different concentration test hydrogels and primary versus murine skin cells (** *p* < 0.01; *** *p* ˂ 0.001; **** *p* < 0.0001).

It was identified that the hydrogels’ action is dose-dependent. In the case of JB6 Cl 41-5a cells (Figure 9B), lower percentages of cytotoxicity were observed compared to human keratinocytes (HaCaT) (Figure 9A), thus, for the CAF-S1-CP940 hydrogel, the values of 15% and 17% were identified, and for the CAF-S2-CP940 hydrogel, values of 11% and 16% were recorded, when both were tested the highest tested concentration (150 μg/mL). The Blank-CP940 and CAF-CP940 samples showed higher values, meaning that these samples did not have cytotoxic action on the tested skin cell lines (Figure 9A,B).

Therefore, after the cell stimulation for 24 h, the obtained results revealed that the carbomer-based hydrogel formulations containing caffeine and phosphorus derivatives do not have a cytotoxic effect on any of the tested healthy skin cell lines (human or murine) because the percentages recorded were up to 20%. This outcome is in agreement with the previous investigation performed. When compared with our previously reported study [65], in which the phosphorus derivatives (S1 and S2) were tested, no significant signs of cytotoxicity were recorded; this affirmation follows the cytotoxic values recorded, which were up to 11%. Nonetheless, the cytotoxic effect was dose-dependent, increasing with the increase of the dosage. However, when tested against the HaCaT cells, the 2-carboxyethylphenylphosphinic acid compound (S2) showed the most pronounced cytotoxic effect, with a percentage rate of 9.03%. The slight difference obtained for the 2-carboxyethyl phenyl phosphinic acid (S2) compound (9.03%) as compared with the carbomer-based hydrogel containing caffeine and 2-carboxyethyl phenyl phosphinic acid (CAF-S2-CP940) (11%) may come either from the concentration of the test sample (5 mM vs. 150 μg/mL) or the caffeine content used in the preparation of the carbomer-based hydrogels. 

#### 2.3.4. Hoechst Staining

**Figure 10:**
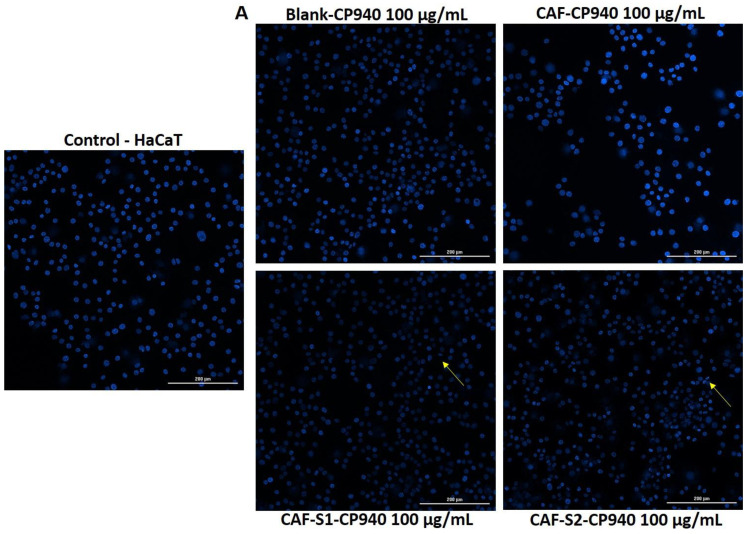

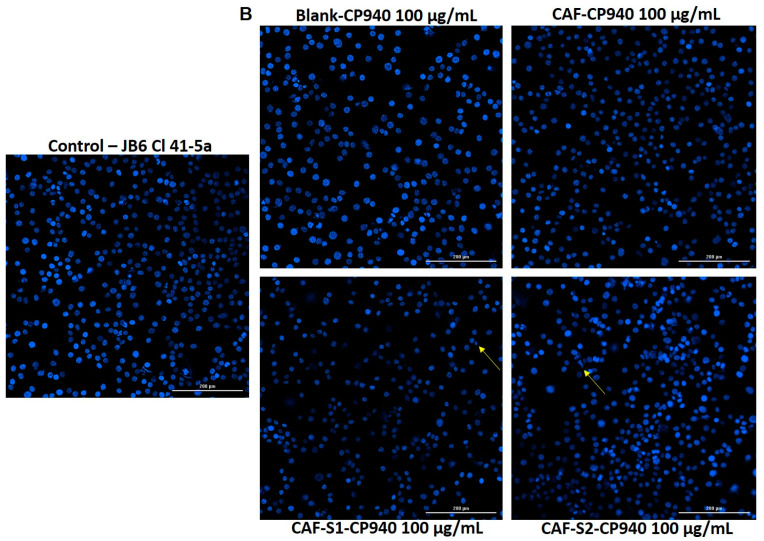
Cell nuclei staining of (**A**) HaCaT and (**B**) JB6 Cl 41-5a cells, at 24 h post-exposure to Blank-CP940, CAF-CP940, CAF-S1-CP940, and CAF-S2-CP940 (100 μg/mL) hydrogels. Yellow arrows mark the apoptosis-related changes. The representative images presented were chosen from one experiment of three. Scale bars represent 200 µm.

To investigate the impact of carbomer-based hydrogel formulations (Blank-CP940, CAF-CP940, CAF-S1-CP940, and CAF-S2-CP940) at the nuclear level, a more specific analysis of what possible damage these formulations could produce against HaCaT (Figure 10A) and JB6 Cl 41-5a (Figure 10B) cell lines was employed by using Hoechst 33342 staining.

The outcomes obtained exhibited that, in the case of the hydrogel formulations based on caffeine and phosphorus derivatives (phenyl phosphinic acid (CAF-S1-CP940), and 2-carboxyethyl phenyl phosphinic acid (CAF-S2-CP940)), a slight reduction in the size of the nuclei appeared when applying the concentration of 100 µg/mL. In addition, the presence of some apoptotic bodies was observed (yellow arrows in Figure 10A,B). However, it cannot be stated that there are signs to support that the two carbomer-based hydrogel formulations containing caffeine and phosphorus derivatives induce apoptosis in the two healthy skin cell lines that were tested. This outcome is consistent with the one obtained in our previous study [65], in which it was reported that HaCaT cells treated with S1 and S2 compounds did not manifest important hallmarks of apoptosis. Moreover, the apoptotic index for the 2-carboxyethyl phenyl phosphinic acid (S2) was above 60% when tested at the highest concentration (5 mM). 

The evaluation of carbomer-based hydrogels containing phosphorus derivatives on human healthy cell lines (HaCaT) and neonatal BALB/c epidermal cells (JB6 Cl 41-5a) holds significant promise for advancing the understanding of their potential applications. Generally, hydrogels have garnered attention for their biocompatibility and potential in wound healing and tissue regeneration. The hydrogel’s biocompatibility at the cellular level is worthy of consideration by studies in which hydrogels showed high cell viability values on human keratinocyte cells (HaCaT) and human fibroblast cells [101]. This underscores the importance of assessing the impact of hydrogels on different cell lines to ensure their safety and efficacy. For instance, a study conducted by Kraskiewicz and co-workers [102] investigated the biological activity of hydrogel-released factors from human adipose tissue-derived mesenchymal stem cells (HATMSC2) on fibroblasts, endothelial cells, and keratinocytes. Kraskiewicz et al.’s study emphasized the importance of assessing the biocompatibility and regenerative potential of hydrogel-released factors, displaying the diverse applications of hydrogel-based therapies in wound healing and tissue regeneration. In another study, the biocompatibility of Manuka-honey-loaded hydrogel scaffolds on human fibroblast and keratinocyte cell lines was assessed by Tomić and his research group [103]. Their findings underscored the influence of honey content on cell viability within the hydrogel scaffolds, indicating the importance of bioactive components in hydrogel formulations for promoting cell health and proliferation. All these studies have shown that the viability of skin human healthy cell lines is influenced by the hydrogel composition, such as the content of Manuka honey, indicating the importance of understanding the interactions between hydrogel components and cells [103]. This emphasizes the need for comprehensive assessments of hydrogel formulations to optimize their biocompatibility for various cell types. 

Regarding the evaluation of hydrogels for wound healing applications, studies have assessed parameters like cell viability and migration potential in human keratinocyte cells (HaCat) [104]. The outcomes provide valuable insights into the effects of hydrogels on HaCaT cell behavior, which are essential considerations in the development of wound healing therapies. Understanding how hydrogels interact with specific cell types like keratinocytes (HaCaT) is crucial for designing effective wound-healing strategies. Furthermore, the preparation of hydrogels crosslinked with compounds like tannic acid has revealed their potential to enhance cell viability. The findings have shown that the hydrogel-based tannic acid stimulates the viability of HaCaT cells as compared to control groups, indicating the beneficial effects of certain hydrogel formulations on cell health [105]. Such findings underscore the importance of exploring novel crosslinking agents to improve the biological properties of hydrogels for various biomedical applications. Based on this statement, the phosphorus derivatives employed in the present study (phenyl phosphinic acid and 2-carboxyethyl phenyl phosphinic acid) were assessed from a biological point of view on HaCaT cells and the results showed that in the case of the S1 compound (phenyl phosphinic acid) the decrease in cell viability was not significant at low concentrations (1 and 2.5 mM), while at high concentration (5 mM) the cell viability was decreased up to about 88%. As regards the S2 compound (2-carboxyethylphenylphosphinic acid), the cell viability was more reduced as compared with the S1 compound, reaching a value of 77% at the same concentration of 5 mM. However, despite all that, we cannot say that compared to the values presented above, the two compounds have a negative impact on HaCaT skin cells [65]. It seems that incorporated into the carbomer-based hydrogel, the two phosphorus derivatives (S1 and S2) present approximately the same values regarding cell viability of HaCaT cells (when tested at the highest concentration (150 μg/mL)) as those reported after testing them at other values (CAF-S1-CP940—81% vs. 88% in the case of S1; CAF-S2-CP940—78% vs. 77% in the case of S2). The slight difference may come from the tested sample concentration (5 mM vs. 150 μg/mL) or from the caffeine content used in the preparation of the carbomer-based hydrogels, which indicates that caffeine used in high concentrations can slightly reduce cell viability. According to this statement, Makhija and co-workers [106] showed that the poly (acrylic acid)-based hydrogel containing 30 mg of *Coffea arabica* extract, induced HaCaT cell viability decreased up to 86% at a tested concentration of 50 μL for 24 h. The authors found that with the increase in the hydrogel application volume, the cell viability of HaCaT cells decreases. Contrary, in another research conducted by Xu et al. [107] the authors reported that caffeine, with a concentration ranging from 128–512 μM, does not affect the HaCaT cell viability at 24 h of exposure, exhibiting viability rates of over 90%. Moreover, Xuan and his research group [108] demonstrate that the film that surrounds the raw coffee bean, named coffee silver skin, can be used as a potential candidate for the cosmetic industry, as a natural functional material in cosmetic products. In addition, in another study conducted by Sarobo and co-workers [109], it has been shown that chronic caffeine intake stimulates epithelial cell proliferation. 

The Carbopol 940 gel has been often used as a vehicle for encapsulating various agents, prepared for delivering bioactive compounds, or as wound healing agents [110,111,112,113]. This synthetic polymer has been selected as a gelation agent due to its complex structure and because it does not cause skin irritation when is used as a carrier for lipid vesicles in transdermal applications. Kirf and co-workers [114] highlight the importance of evaluating the toxicity profile of hydrogel components to ensure their suitability for biomedical uses, including wound healing. The authors have developed N-vinyl-2-pyrrolidone–acrylic acid-based copolymeric hydrogels and performed cyto- and genotoxicological assessments on human keratinocytes (HaCaT), demonstrating their non-cytotoxic effect on human skin cell lines. 

Overall, a comprehensive toxicological screening of carbomer-based hydrogel formulations containing caffeine and phosphorus derivatives offers valuable insights into their safety profiles and potential risks associated with their use. Therefore, the findings of this study not only contribute to the current knowledge on the safety of carbomer-based hydrogels containing caffeine and phosphorus derivatives but also inform future research directions aimed at optimizing the design and development of safe and effective hydrogel formulations for various applications in pharmaceutical and cosmetic industries.

### 2.4. Gene Expression

In the context of human immortalized keratinocyte cells (HaCaT), which are commonly used in skin research, real-time RT-PCR can provide detailed insights into how carbomer-based hydrogels influence the expression of specific genes involved in inflammation, cell proliferation, or other relevant pathways [115,116]. By comparing gene expression levels in HaCaT cells treated with carbomer-based hydrogels containing caffeine and phosphorus derivatives to untreated cells, we can determine the precise effects of these hydrogels at the molecular level, aiding in understanding the mechanisms underlying observed biological responses. To ensure data reliability and reproducibility both carbomer-based hydrogel formulations containing caffeine and phosphorus derivatives were tested in triplicate across independent experiments. For the gene expression, the quantitative analyses will be further normalized to the *18S* housekeeping gene [117,118,119]. The significant changes that occurred in gene expression, will be statistically analyzed (by a level of significance (*p* < 0.05)), through comparison with the untreated control group [120,121,122].

The results demonstrate significant differences in gene expression for Caspase 3 and Bax when comparing control and CAF-S2-CP940 treatments, with *p*-values of 0.048 and 0.030, respectively. However, comparisons between control and CAF-S1-CP940 treatments for all genes did not yield significant results, suggesting that CAF-S1-CP940 does not significantly alter gene expression compared to the control. Additionally, there were no significant changes in the expression of *Bcl-XL*, *BAD*, *Bcl-2*, and *Caspase-8* genes with CAF-S2-CP940 treatment, as indicated by their non-significant *p*-values (Table 2).

The gene expression analysis in HaCaT cells treated with carbomer-based hydrogel formulations containing caffeine and phenyl phosphinic acid (CAF-S1-CP940) and 2-carboxyethyl phenyl phosphinic acid (CAF-S2-CP940) reveals significant differences in their biological impacts, particularly on apoptotic and cell survival pathways [123,124,125]. By normalizing to the *18S* housekeeping gene, the fold change in expression levels of several key genes was calculated. The treatment with CAF-S2-CP940 hydrogel generally resulted in more pronounced changes in gene expression compared to CAF-S1-CP940 hydrogel. Specifically, the genes associated with apoptosis, such as *Caspase 3*, and *Bax*, showed substantial upregulation under CAF-S2-CP940 hydrogel treatment. This indicates a stronger pro-apoptotic response, suggesting that CAF-S2-CP940 hydrogel is more effective at inducing apoptosis in HaCaT cells. For instance, the fold change for *Caspase 3* was approximately 1.37 under CAF-S2-CP940 hydrogel treatment, compared to a decrease in expression after treatment with CAF-S1-CP940 hydrogel. Similarly, *Bax* exhibited a significant increase with a fold change of 1.40 under treatment with CAF-S2-CP940 hydrogel [116,126].

In contrast, CAF-S1-CP940 hydrogel treatment generally resulted in minimal changes or slight decreases in the expression of these apoptotic genes. The fold change for most genes under CAF-S1-CP940 hydrogel treatment hovered around 0.83, indicating a slight decrease in expression. This suggests that the hydrogel containing caffeine and phenyl phosphinic acid (S1) has a less pronounced impact on promoting apoptosis compared to the hydrogel containing a mixture of caffeine and 2-carboxyethylphenylphosphinic acid (S2). Moreover, genes involved in cell survival mechanisms, such as BCL-XL and Bcl-2, also showed differential expression. Under treatment with CAF-S2-CP940 hydrogel, BCL-XL, and Bcl-2 were significantly upregulated, with fold changes of 1.37 and 1.38, respectively. This upregulation suggests that the hydrogel based on caffeine and 2-carboxyethylphenylphosphinic acid (S2) enhances anti-apoptotic pathways, potentially contributing to improved cell survival [120].

Overall, the data indicates that CAF-S2-CP940 hydrogel exerts a stronger influence on both promoting apoptosis and enhancing cell survival mechanisms in HaCaT cells compared to CAF-S1-CP940 hydrogel. These findings highlight the potential of 2-carboxyethylphenylphosphinic acid (S2) as a more effective therapeutic agent, capable of inducing apoptosis while also promoting cell survival through the upregulation of key anti-apoptotic genes. Further research is warranted to explore the clinical relevance and underlying mechanisms of these observations, paving the way for potential new treatments in oncological and dermatological applications.

### 2.5. In Ovo Toxicological Screening of Carbomer-Based Hydrogel Formulations

To complete the safety profile of carbomer-based hydrogel formulations containing caffeine and phosphorus derivatives, the evaluation of the in ovo anti-irritant effect was performed using the HET-CAM assay. For the evaluation of the possible irritant effect of hydrogel samples, a concentration of 100 μg/mL was selected for both test samples composed of a mixture of caffeine and phosphorus derivatives. The HET-CAM (Hen’s Egg Test on the Chorioallantoic Membrane) method was utilized to assess any vascular changes induced by the hydrogels, such as hemorrhage, coagulation, or lysis. As a positive control, sodium lauryl sulfate 1% (SLS) was selected, and as a negative control, purified distilled water was chosen. The irritation score (IS) was calculated for each sample and control group based on the occurrence of lysis, hemorrhage, and coagulation processes using the formula provided in the materials and methods section. The results, as presented in Table 3, demonstrated that the positive control exhibited a substantial irritant effect, with an irritation score of 18.070, while the negative control was not irritant at all, with an irritation score of 0.070. 

**Figure 11:**
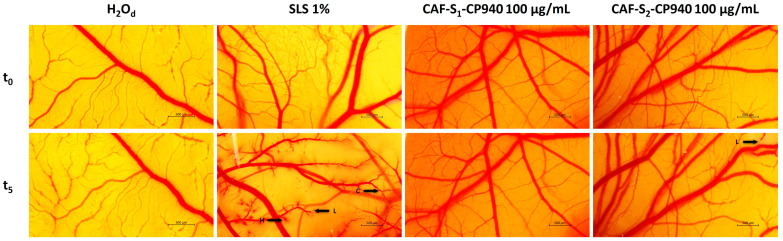
Representative images of the irritant potential of CAF-S1-CP940 and CAF-S2-CP940 by the HET-CAM method. Stereomicroscopic images of the chorioallantoic membrane after treatment with H_2_Od (negative control), SLS (positive control), and test samples at a concentration of 100 μg/mL. The arrows indicate areas with the occurrence of lysis (L), hemorrhage (H), and coagulation (C).

Application of the samples and controls to the chorioallantoic membrane revealed hemorrhage, lysis, and coagulation in the positive control group, with only slight lysis in the case of sample CAF-S2-CP940—sample containing a mixture of caffeine and 2-carboxyethyl phenyl phosphinic acid (irritation score of 0.304), while neither of these processes was observed in the negative control or sample CAF-S1-CP940—sample containing a combination of caffeine and phenyl phosphinic acid (irritation score of 0.070). These findings suggest that hydrogel CAF-S1-CP940 has an even lower irritant potential compared to hydrogel CAF-S2-CP940, but both of them are non-irritant compounds (Figure 11).

The HET-CAM assay, which stands for the Hen’s Egg Test on Chorioallantoic Membrane, is a valuable assay for evaluating the effects of hydrogel formulations. This assay offers versatility in assessing different substances, including those insoluble in water or solids [127,128]. This versatility is particularly important when testing the effects of hydrogels, which are complex mixtures of oils and structuring agents. The HET-CAM assay is also known for its speed and simplicity, making it a feasible alternative for evaluating potential irritant properties of formulations [127]. Furthermore, the assay is sensitive and cost-effective, requiring no special equipment, which is advantageous for routine testing of various chemicals and formulations [129]. By utilizing the HET-CAM assay, researchers can efficiently assess the cytocompatibility and potential irritant properties of hydrogel formulations, providing valuable insights into their safety and efficacy for various applications.

Our findings regarding the in ovo study demonstrate that the hydrogels evaluated at a concentration of 100 µg/mL exhibited a high level of safety on the chorioallantoic membrane. Specifically, the application of hydrogel CAF-S1-CP940 did not result in any hemorrhage, coagulation, or lysis processes, yielding an irritation score of 0.070, which was equivalent to the negative control, purified distilled water. Conversely, the application of hydrogel CAF-S2-CP940 induced minimal lysis only in the final 10 s of the experiment, with an irritation score of 0.304. Both formulations of hydrogels can be classified as safe for topical use, as they fall within the non-irritant category based on the irritation scale proposed by Luepke (ranging from 0 to 0.9), with their irritation scores below the threshold of 0.9 [130].

The exceptional biocompatibility and unique attributes of hydrogels for topical applications underscore their potential as compelling subjects for extended scrutiny through in-ovo assays. This avenue of research holds promise for yielding valuable insights into the multifaceted impacts of hydrogels on various biological mechanisms. Moving forward, future studies could delve deeper into exploring the specific molecular interactions between hydrogels and the chorioallantoic membrane, elucidating the underlying mechanisms that contribute to their observed safety and efficacy profiles. Additionally, investigating the long-term effects of repeated exposure to hydrogels in in ovo models could provide a more comprehensive understanding of their biocompatibility and potential applications in therapeutic interventions. Such investigations would not only advance our knowledge about hydrogels but also pave the way for their optimized utilization in diverse biomedical and pharmaceutical contexts.

### 2.6. In Vivo Toxicological Screening of Carbomer-Based Hydrogel Formulations

Animal models are vital for biomedical research, providing a bridge between basic science and clinical application. They enable the study of disease pathologies, support drug development, and improve surgical techniques, among other contributions. On the other hand, to evaluate several skin parameters (e.g., skin pH, trans-epidermal water loss (TEWA), melanin and erythema, the degree of stratum corneum moisture, sebum, etc.), contemporary and non-invasive approaches were employed.

The evaluation of trans-epidermal water loss (TEWL), erythema, and skin hydration levels is crucial for understanding the effects of a compound after topical applications on healthy skin. Therefore, to assess the effect of carbomer-based hydrogels containing phosphorus derivatives as biocompatible and safe formulations with healthy skin, an in vivo experiment was performed using BALB/c nude mice (hairless mice) as an experimental model for this purpose. The evolution of the above-mentioned skin parameters after topical application was followed, as well as eventual body weight loss and behavioral patterns. To establish a comprehensive toxicological profile it is crucial to understand the impact of these formulations on healthy skin. 

The experimental model used in the current study was adult male BALB/c hairless mice. No significant modifications were observed in any groups of mice as regards their body weights, recorded every 3 days for 15 days. Moreover, there were observed no signs of behavioral patterns (salivation, lethargy, sleep, and coma) during the experiment. These outcomes indicate that skin applications of carbomer-based hydrogel formulations containing caffeine and phosphorus derivatives are not related to weight loss or negative effects at the nervous system level. The mice’s skin appearance was another parameter assessed after topical application of the carbomer-based hydrogel formulations containing caffeine and phosphorus derivatives. The modifications regarding skin appearance were described by monitoring the evolution of mice’s skin physiological parameters—skin hydration, erythema, and trans-epidermal water loss. The skin measurements were recorded before the topical application of the carbomer-based hydrogels (these values were considered the reference values) and after each topical application (at 20 min after application). Each application occurred on every third day until the last day of the experiment (day 15). 

**Figure 12:**
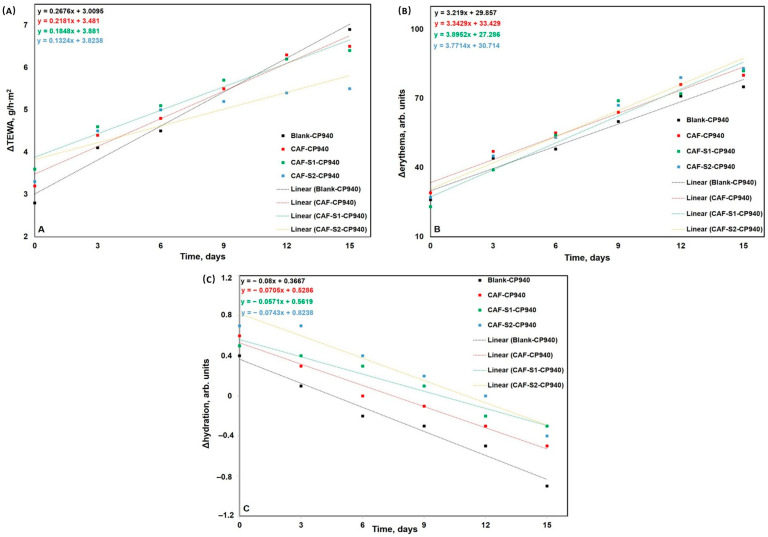
Comparative evolution of mice’s skin biophysical parameters: (**A**) Trans-epidermal water loss (TEWA)-related measurements; (**B**) Erythema-related measurements; (**C**) Hydration-related measurements. The experiments were performed in triplicate and the mean value ± SD was calculated from three individual experiments. The one-way ANOVA statistical analysis followed by Tukey’s multiple comparison post hoc test was applied to evaluate the significant differences that occurred between carbomer-based hydrogel formulations containing caffeine and phosphorus derivatives (CAF-CP940; CAF-S1-CP940; CAF-S2-CP940) and Blank-CP940.

The primary markers of any potential irritant impact given by the carbomer-based hydrogels are the changes in TEWA, erythema, and skin hydration levels, which are shown in Figure 12A–C. As regards the changes in trans-epidermal water loss, a similar trend of TEWA (slight increases) was found for all 4 groups of hydrogels (Figure 12A). The slight difference between the increase of TEWA for hydrogels and Blank-CP940 implies that these products did not significantly alter the TEWA compared to the Blank-CP940 and they do not have a discernible impact on the skin’s barrier function. It is worth mentioning that the last differences that were obtained after 15 days are lower for hydrogels than for Blank-CP940; it seems that the obtained samples play as protective agents for the skin. Comparing the two hydrogels containing caffeine and phosphorus compounds (phenylphosphinic acid—CAF-S1-CP940 and 2-carboxyethylphenylphosphinic acid—CAF-S2-CP940) it seems that the latter shows a slightly increased protective effect for mice’s skin than the first (Figure 12A). 

When considering the impact of carbomer-based hydrogels on skin parameters in BALB/c nude mice, it is essential to refer to studies that focus on similar evaluations in murine models to draw parallels and extrapolate potential outcomes. In the literature, research studies that have assessed TEWL, erythema, and skin hydration provide valuable insights into the skin barrier function, inflammation, and moisture levels [131,132,133]. TEWL measurement is a particularly important noninvasive method as it reflects the water loss through the epidermis, indicating the integrity of the skin barrier [131]. High TEWL values may indicate increased water loss through the mice’s skin, suggesting a compromised barrier function [133]. Monitoring changes in TEWL over time following the application of these carbomer-based hydrogels can help in understanding their impact on mice’s skin hydration and barrier integrity. Bukowska and co-workers [134] explore the effect of various factors on dermal fibroblasts derived from Balb/c mice. Dermal fibroblasts are crucial for skin hydration and barrier function, and further investigations of the functional characteristics of these cells after carbomer-based hydrogel applications can provide valuable insights into the hydrogels’ impact on skin parameters at a cellular level.

Erythema, characterized by redness of the skin, is probably the most important parameter used to assess the irritation potential of compounds, including chemicals, pharmaceuticals, and cosmetic products. Its evolution can indicate the severity and progression of the irritant response, providing valuable information for safety assessments (Figure 12B). Changes in erythema differences (Figure 12B) present upward trends for all four groups of compounds, which are normal for such a sensitive skin parameter. It is important to mention that the amplitude of these changes is limited to approx. 50 units/15 days; as a comparison, the application of pure chili pepper extract on the skin of the same mice strain resulted in around 100 units during the same period [135]. According to the literature’s data, skin redness, assessed through the evaluation of erythema, can be indicative of inflammation or irritation [136,137]. This parameter is crucial for assessing the skin’s response to the topical treatment, ensuring that the topical compound tested does not cause adverse reactions. 

It is well known that the potential skin irritation assessment represents a new alternative method to evaluate the hazard of a newly developed compound. The skin sensitivity parameter is used in many toxicity assays, usually on newly developed cosmetic products, but it can also be used to evaluate any chemical substance. Measuring skin hydration levels after applying hydrogels can provide valuable information on the moisturizing effects of these formulations [138,139]. Figure 12C shows downward trends of hydration levels that are often correlated with the increases of TEWA parameter; however, these decreases are not significant and they are smaller in the case of hydrogels CAF-CP940, CAF-S1-CP940, and CAF-S2-CP940 than the decrease of hydration level recorded for Blank-CP940, which was around 1.2 units/15 days.

The skin hydration assessment is essential for determining the moisture levels in the skin, which is vital for skin health and function [138,139,140]. Because skin hydration is essential for maintaining skin health, a decrease in hydration levels can lead to dryness and skin barrier impairment. By assessing changes in skin hydration following the topical applications of hydrogels, researchers can determine their efficacy in improving skin moisture levels and overall skin health. 

In conclusion, evaluating skin parameters such as TEWL, erythema, and skin hydration levels after the topical application of carbomer-based hydrogels containing phosphorus derivatives on BALB/c nude mice is essential for understanding the effects of these formulations on skin physiology. By utilizing these parameters, researchers can assess the impact of the hydrogels on skin barrier function, inflammation, and moisture levels, providing valuable insights into their potential benefits for skin health.

## 3. Conclusions

In the present study, 1 wt% Carbopol 940-based formulations containing 0.5 wt% caffeine and phosphorus derivatives (phenylphosphinic acid, and 2- carboxyethylphenylphosphinic acid (0.5 M)) as active ingredients were successfully prepared without the usual carbomer neutralization with a base. The biological profile of all four developed carbomer-based hydrogel formulations indicated a good biosafety profile when both the HaCaT and JB6 Cl 41-5a cells were exposed at the maximum concentration tested of 150 μg/mL, for 24 h. Although a slight decrease in cell viability was observed on both cell lines. When tested at the highest concentrations, viability did not drop below 70%. We cannot affirm that the two developed hydrogels harm healthy skin cells. Moreover, on JB6 Cl 41-5a cells, the CAF-S2-CP940 hydrogel (sample containing a combination of caffeine and 2-carboxyethylphenylphosphinic acid) led to a slight cell proliferation when the first two concentrations were tested. Overall, the CAF-S2-CP940 hydrogel was more active in the in vitro tests on HaCaT cells as compared to the CAF-S1-CP940 hydrogel (sample containing a combination of caffeine and phenylphosphinic acid). Analyzing the expression levels of multiple genes simultaneously, one can affirm that the gene expression encompasses cell viability, cytotoxicity, and morphological changes, allowing to comprehensive understanding of the overall impact of carbomer-based hydrogels on the genetic profile of HaCaT cells. To complete the toxicological profile, in-vivo biocompatibility and in ovo toxicity tests were performed, and the outcomes revealed that both developed hydrogels based on carbomer containing caffeine and phosphorus derivatives exerted good biocompatibility after topical application on mice’s skin and a lack of toxicity after exposing the hen’s chicken embryo to the hydrogel formulations. In summary, by corroborating the findings presented in the current study, one can affirm that the biosafety profile of carbomer-based hydrogel formulations containing caffeine and phosphorus derivatives was demonstrated. Despite all, further investigations regarding their therapeutic efficacy as well as stability studies, formulation validation, and safety on long-term use are needed for potential clinical translatability. 

## 4. Materials and Methods

### 4.1. Materials

The active ingredients used for hydrogel preparation were as follows: caffeine (CAF) purchased from Acros Organics (Geel, Belgium); phenylphosphinic acid (S1) was acquired from Sigma Aldrich Merck KgaA (Darmstadt, Germany), and 2- carboxyethyl phenylphosphinic acid (S2) was obtained by our group of research, being characterized, and investigated. The outcomes obtained were reported in a previously published work [65]. 

Hydroxypropylmethylcellulose (from Benecel^TM^ E4M PHARM, Ashland, Rotterdam, The Netherlands), chitosan (from Chitopharm^®M^, S.C. Antibiotice S.A. Iaşi, Romania), carbomer (Carbopol 940, from Lubrizol Advanced Materials, Brussels, Belgium) and colloidal silicon dioxide (from Aerosil 200 Pharma, Evonik Industries AG, Hanau, Germany) were received as gift samples.

**Figure 13:**
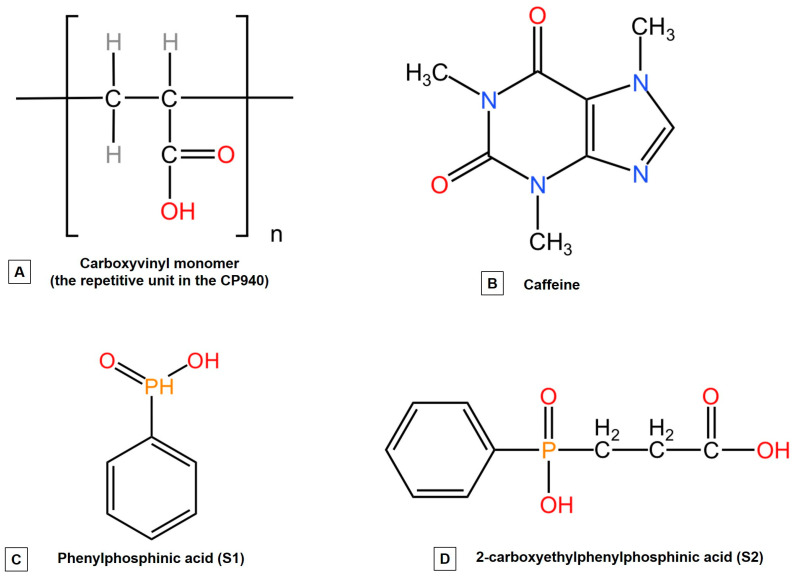
Chemical structures of the gelling agent (**A**) Carbopol 940 (CP940), as well as of the active ingredients for carbomer-based hydrogel formulations preparations: (**B**) Caffeine, (**C**) phenylphosphinic acid (S1), and (**D**) 2-carboxyethylphenylphosphinic acid (S2). The chemical structures were generated using KingDraw software version 1.1.0.

The chemical structures of the active ingredients used for the synthesis of carbomer-based hydrogel formulations as well as the chemical structure of the gelling agent (Carbopol 940) are presented in Figure 13. 

For the in vitro experiments, the specific culture media used for cell growth were Dulbecco’s modified Eagle medium (DMEM), Eagle’s Minimum Essential Medium (EMEM), fetal bovine serum (FBS) as cell culture supplement, a solution based on trypsin and EDTA, and the MTT (3-(4,5-dimethylthiazol2-yl)-2,5-diphenyltetrazolium bromide) viability kit (Sigma Aldrich, Steinheim, Germany). A mixture based on Penicillin and streptomycin (of 10,000 IU/mL), phosphate saline buffer (PBS), as well as dimethyl sulfoxide (DMSO-solvent), were procured from Aidenbach, Germany—PAN-Biotech GmbH. ThermoFisher Scientific (Waltham, MA, USA) provided the lactate dehydrogenase (LDH) kit as well as the Hoechst 33342 dye.

The cell cultures used in the present study were the immortalized human keratinocytes (HaCaT) and the neonatal BALB/c epidermal cells (JB6 Cl 41-5a). The American Type Culture Collection (Manassas, VA, USA) provided both cell lines. The HaCaT cells were cultured in DMEM culture medium, while the JB6 Cl 41-5a cells were cultured in EMEM medium, extra supplemented with FCS (10%—for DMEM and 5% for EMEM) and 1% antibiotic mixture (penicillin-streptomycin), followed by maintaining under specific conditions (5% CO_2_ and 37 °C).

### 4.2. Animals

To evaluate the biosafety profile of carbomer-based hydrogel formulations containing caffeine and phosphorus derivatives on healthy skin, an in vivo experiment was performed. The experimental protocol and procedure were established according to the protection of animals for scientific research, taking into account the European Directive 2010/63/EU and the National Law 43/2014. Before the experiment started, ethics approval was obtained from the Committee for Research Ethics of “Victor Babes” University of Medicine and Pharmacy, Timisoara, Romania (No. 93 from 21.11.2022). Throughout the experiment, the animals received water and food and were kept in plastic cages in the University animal facility. The standard condition for the livelihood of animals consists of a constant temperature of 22.5 ± 2 °C and relative humidity of 55% ± 5%, with a cycle of 12 h/12 h (light/dark). Before 2 weeks of the experiment, the animals were acclimatized to laboratory conditions. The animals used in the present study were twelve healthy adult male BALB/c nude mice (hairless mice) that were 7 weeks old (weight = 16 ± 2 g) at the time of this study. The animals were procured from Charles River, Sulzfeld, Germany. 

### 4.3. Methods

#### 4.3.1. The Synthesis of Carbomer-Based Hydrogel Formulations

**Figure 14:**
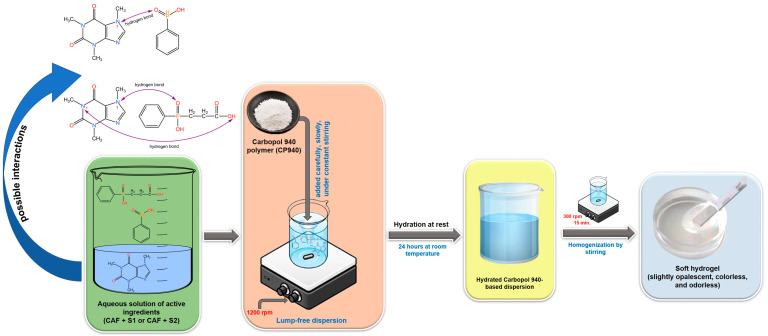
Carbomer-based hydrogel formulations schematic protocol.

To obtain carbomer-based hydrogel formulations containing caffeine (sample denoted CAF-CP940) or its mixtures with phosphorus derivatives (samples denoted CAF-S1-CP940 and CAF-S2-CP940), the Carbopol 940 polymer (CP940) was selected as the appropriate gelling agent. The carbomer-based hydrogels containing bioactive compounds (caffeine 0.5 wt%, phenylphosphinic acid 0.5 M, and 2-carboxyethylphenylphosphinic acid 0.5 M), were prepared based on the procedure recommended by the manufacturer [141]. Thus, the CP940 was dispersed carefully and slowly in the aqueous solution containing active ingredients at room temperature under constant stirring at 1200 rpm, using a laboratory overhead stirrer (Eurostar Digital IKA Werke, Staufen, Germany). After the addition of the CP940 polymer powder, the stirring was continued at 1200 rpm for 15 min, and then the obtained dispersion was left at rest for 24 h. Finally, to homogenize the systems, the mixtures were further stirred at 300 rpm for 15 min. The CP940 polymer concentration in the hydrogel formulations was 1 wt%. To prepare blank carbomer-based hydrogel (sample denoted Blank-CP940); the specific amount of CP940 polymer was homogenously dispersed into a known amount of distilled water at room temperature, using the same procedure described above. The schematic protocol for the synthesis of carbomer-based hydrogel formulations is depicted in Figure 14. 

**Figure 15:**
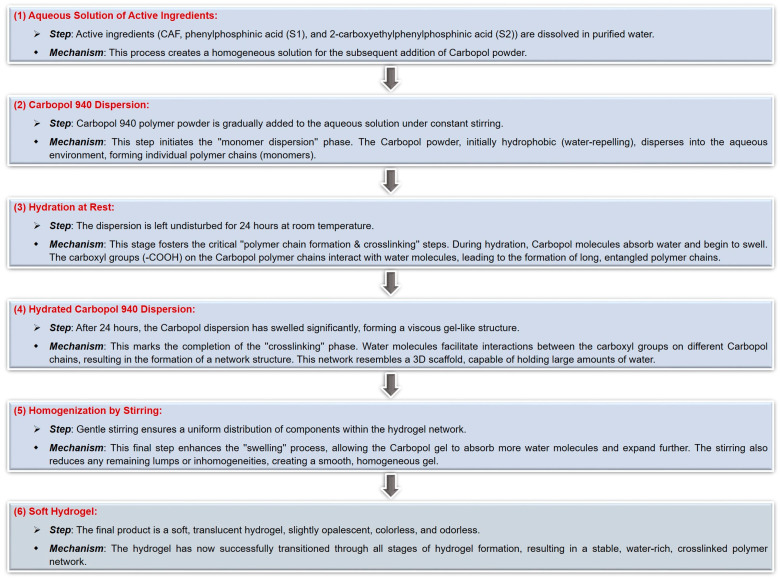
Block diagram containing the possible mechanism of carbomer-based hydrogel formation.

In addition, because hydrogel formation is a multifaceted process that involves various mechanisms depending on the specific composition and intended application of the hydrogel, a step-by-step mechanism of carbomer-based hydrogel formation was proposed (Figure 15).

Through the optimized structures analysis and interaction energies of the active ingredients (CAF, phenylphosphinic acid (S1), and 2-carboxyethylphenylphosphinic acid (S2)), the first step is to identify the preferred binding positions for future determination of the key contributions to the intermolecular interactions. It is believed that phenylphosphinic acid (S1) binds to caffeine through a combination of hydrogen bonding and van der Waals interactions based on group binding affinity. Perhaps the most preferred binding position involves the P=O group of the S1 compound forming a hydrogen bond with the N7 atom of caffeine (possible interactions presented in Figure 14). This interaction could be further stabilized by van der Waals contacts between the phenyl ring of both phosphorus derivatives and the methyl groups of caffeine. In the case of 2-carboxyethyl phenyl phosphinic acid (S2), due to its additional carboxyethyl group, this compound could exhibit a more complex binding pattern with caffeine. The carboxyl group of the S2 compound could interact with the N1 atom of caffeine through a hydrogen bond, while the P=O group could form a hydrogen bond with the N7 atom similar to the S1 compound. The phenyl ring of the S2 compound could also interact with the methyl groups of caffeine through van der Waals forces. Based on the chemical structures presented in Figure 13, one can observe that the S2 compound has a carboxyethyl group, which could provide additional contact points for interactions, thus leading to a stronger binding affinity compared to the S1 compound. This initial stage sets the foundation for the structural integrity of the hydrogel. 

Subsequently, the gelation process occurs and the gel passes through different stages such as an increase in viscosity, clusters formation within the gel matrix, and finally, the formation of the hydrogel as critical concentrations of components are reached [142]. This step-by-step progression highlights the gradual transition from a liquid to a gel state, indicating the development of the hydrogel matrix. 

#### 4.3.2. Characterization of Carbomer-Based Hydrogel Formulations

##### Determination of Macroscopic Properties and pH 

Macroscopic evaluation of carbomer-based hydrogel formulations was performed 24 h after preparation, by visual assessment of their organoleptic properties (physical appearance, transparency, color, and homogeneity) [67].

The pH of experimental hydrogel formulations was performed in triplicate using a calibrated SevenExcellence™ S400-KIT pH-meter (Mettler Toledo, Columbus, OH, USA) [143]. Briefly, 1 g of each formulation was mixed with 20 mL of purified water and stirred for several minutes until a dispersion was obtained. Further, the dispersion was filtered and the pH was measured. Data were expressed as mean ± SD. 

##### Rheological Measurements

The rheological features (viscosity and flow behavior) of the carbomer-based hydrogel formulations were investigated by the steady-state flow test, using a HAAKE RheoStress 1 rheometer (Thermo Fisher Scientific, Karlsruhe, Germany) with temperature device controller (TCP/P Peltier /Plate), at rotational mode. A slightly modified protocol previously described by Coneac and co-workers was applied [81]. Briefly, it used a cone-plate geometry (d = 35 mm), a cone angle of 1° (C35/1°), and a gap size of 0.05 mm, at a temperature of 23 °C (under the Peltier system control). The flow behavior and viscosity of the carbomer-based systems were determined. The parameters set up for the flow curves and viscosity curves were a share rate over 0.05–100 s^−1^, which increases progressively for 120 s; a constant share rate period of 10 s at 100 s^−1^ followed by a ramp-down period for another 120 s. Using HAAKE RheoWin 4 software version 4.3 (Thermo Fisher Scientific, Karlsruhe, Germany) the rheological data were analyzed. The experiment was performed in triplicate for each measurement. 

##### Spreadability Test

Based on the parallel-plate method [13], the spreadability test was performed. Briefly, 1 g of the sample was applied over squared paper (within the circle marked on a glass plate placed in advance). The second calibrated glass plate was added to the hydrogel for 1 min. Further, several weights of the upper plate (50, 100, 200, 250, 500, and 750 g) were added at 1-min intervals between them. Finally, the spreading area was measured and the results were expressed as the spreading area (mm^2^) of the applied weight. The experiment was performed in triplicate for each measurement, and the mean value ± SD for 750 g standard weight was presented.

##### Scanning Electron Microscopy (SEM Analysis)

The surface morphology of the carbomer-based hydrogel formulations that contained caffeine and phosphorus derivatives was carried out by scanning electron microscopy (SEM) right after the hydrogel matrix was formed. This was performed in a low vacuum mode (LV) with an accelerating voltage of 30.0 kV on a Hitachi SU8230 cold field emission gun STEM microscope from Chiyoda (Tokyo, Japan). The microscope was equipped with secondary electron detectors, upper and lower (EDX detectors X-Max^N^ 80 from Oxford Instruments, Abingdon, UK). The SEM images were recorded at two magnification orders—one for an image overview and the other for higher surface topography, to visualize the region’s surface interest. Each carbomer-based hydrogel formulations were placed on a copper grid support and left to dry for several hours [144]. The dried hydrogels were sputter-coated with carbon (Agar Automatic Sputter Coater (Stansted, Essex, UK)), to ensure better conductivity as well as to obtain high-resolution imaging. 

#### 4.3.3. In Vitro Assays

##### Cell Viability Test 

Carbomer-based hydrogel formulations were first evaluated for their safety on healthy human/murine skin cell lines. The analysis was performed using the MTT colorimetric test to evaluate the action of hydrogels on cell viability. Briefly, the HaCaT and JB6 Cl 41-5a cells were cultured in 96-well plates (1 × 10^4^ cells/well), left to adhere to the bottom of the well, and treated with increasing concentrations (50, 100, and 150 μg/mL) of Blank-CP940, CAF-CP940, CAF-S1-CP940, and CAF-S2-CP940 for 24 h. When the treatment was ended, 10 μL of MTT reagent was added. This was followed by cell incubation (3 h). Further, 100 μL of solubilization buffer was added and left in contact with cells at room temperature for another 30 min. Using a Cytation 5 device from BioTek Instruments Inc. (Winooski, VT, USA), the absorbance level of each well was read at 570 nm to determine the rate viability. The control group was represented by the cells untreated with test samples, treated only with specific growth media. Based on the calculation formula reported in one of our previous articles [145], the cell viability percentages were calculated. The experiments were performed in triplicate. 

##### Cell Morphology, Confluence, and Number Evaluation

The cellular morphology of HaCaT and JB6 CCl 41-5a cells were examined after the treatment with carbomer-based hydrogel formulations containing caffeine and phosphorus derivatives (50, 100, and 150 μg/mL), after 24 h, based on the same principle as it was described in our previous work [146]. The micrographs were taken with an inverted microscope under bright field lighting conditions (Olympus IX73 from Olympus Corporation, Tokyo, Japan), at 20× magnification, using the cellSens Dimensions v.1.8. Software. For the cell confluence and number, the pictures were taken using the Lionheart FX automated microscope, at 4× magnification, after 24 h, and further analyzed using the software Gen5 Microplate Data Collection, version 3.14 (from BioTek Instruments Inc., Winooski, VT, USA).

##### Cytotoxicity Assay via the LDH Release Method

To assess the cytotoxic activity induced by the carbomer-based hydrogel formulations containing caffeine and phosphorus derivatives, at different concentrations (50, 100, and 150 μg/mL), on HaCaT and JB6 Cl 41-5a cells, the lactate dehydrogenase release (LDH) method was employed. Gheran and co-workers [147] described the protocol used. The first step consists of seeding the cells (in a 96-well plate), followed by treatment for 24 h, but only when the confluence of cells reaches 85–90%. Three concentrations of each carbomer-based hydrogel (Blank-CP940, CAF-CP940, CAF-S1-CP940, and CAF-S2-CP940) at quantities 50, 100, and 150 μg/mL were tested. After this treatment, 50 µL of LDH release was added to each well plate, and right after another 50 μL of the reaction mixture was added and the wells were incubated for 30 min in the dark. Finally, 50 μL/well of stop solution kit (LDH assay from Thermo Fisher Scientific, Boston, MA, USA) was added. By using a Cytation 5 device from BioTek Instruments Inc. (Winooski, VT, USA), absorbance levels were read at two specific wavelengths (490 and 680 nm). 

##### Hoechst Nuclear Staining

To determine if the developed carbomer-based hydrogel formulations with caffeine content and phosphorus derivatives could induce cell death through apoptosis or necrosis, the Hoechst nuclear staining test was performed, according to the protocol described in detail in our previous work [146]. The Hoechst 33342 staining test highlights the cytotoxic effects of the hydrogels on the nuclei of healthy human keratinocyte cells (HaCaT) and healthy murine neonatal epidermal cells (JB6 Cl 41-5a). Both cell lines (10^5^ cells/well) were treated with carbomer-based hydrogel formulations (Blank-CP940, CAF-CP940, CAF-S1-CP940, and CAF-S2-CP940) at a concentration of 100 μg/mL for 24 h. After this period, 500 μL/well (1:2000 in PBS) of Hoechst solution was added and the plate wells were again incubated in the dark for several minutes. Finally, both plate wells were washed three times with PBS after the staining solution was eliminated. The fluorescent images were taken under the Lionheart FX automated microscope at a magnification of 10×. This was followed by image processing using the software Gen5 Microplate Data Collection.

#### 4.3.4. RT-qPCR

To investigate the impact of the carbomer-based hydrogels containing caffeine and phosphorus derivatives (CAF-S1-CP940 and CAF-S2-CP940), the real-time reverse transcription–polymerase chain reaction (RT-PCR) method was employed. The RT-PCR is a molecular biology technique suitable for detecting low-abundance mRNA and monitoring amplification products used to quantify gene expression levels in various cell types [115,148]. The impact of each compound on the expression levels of the genes (*BAD*, *BCL-XL*, *Bax*, *Bcl-2*, *Caspase 3*, and *Caspase 8*), was determined after a concentration of 5 mM/compound was applied to HaCaT cells. 

##### RNA Extraction and Quantification

After the HaCaT cells were treated with the test compounds, RNA was extracted by using the Quick-RNA Miniprep Kit (Zymo Research, Irvine, CA, USA), according to the manufacturer’s specifications. By using the DS-11 spectrophotometer (DeNovix, Wilmington, DE, USA), the extracted RNA was investigated as concern its concentration and purity [149].

##### cDNA Synthesis and RT-qPCR

By employing the Maxima^®^ First Strand cDNA Synthesis Kit (Thermo Fisher Scientific, Inc., Waltham, MA, USA), reverse transcription of extracted RNA to cDNA was performed according to the manufacturer’s guidelines. Further, using the system Quant Studio 5 real-time PCR from Thermo Fisher Scientific Inc. (Waltham, MA, USA), a quantitative real-time PCR (RT-qPCR) was carried out. The Power SYBR-Green PCR Master Mix system from Thermo Fisher Scientific Inc. (Waltham, MA, USA) was used for the amplification of specific gene sequences [149].

##### Gene Targets and Analysis

To ensure targeted amplification, primer sequences were used that were specific to the following genes: *BAD*, *BCL-XL*, *Bax*, *Bcl-2*, *Caspase 3*, and *Caspase 8*. Against the housekeeping gene *18S*, the expression levels of the genes mentioned above were normalized [118]. Thermo Fisher Scientific Inc. (Waltham, MA, USA) provided the primers’ oligonucleotides, and the sequences used are presented in Table 4.

#### 4.3.5. In Ovo Assay

To assess the irritant potential of carbomer-based hydrogel samples, containing caffeine and phosphorus derivatives (CAF-S1-CP940 and CAF-S2-CP940), the HET-CAM method was employed following a protocol adapted from ICCVAM [Interagency Coordinating Committee for the Alternative Methods Validation (ICCVAM), ICCVAM Recommended Test Method Protocol: Hen’s Egg Test-Chorioallantoic Membrane 2010, Available online: http://iccvam.niehs.nih.gov/ (accessed on 21 May 2024)]. Fertile hen eggs were incubated under controlled conditions (65% humidity and 37 °C). On the fourth day of incubation, 8 mL of egg white was extracted, and on the fifth day, a window was created to visualize the embryo. Subsequently, the eggs were sealed with medical tape. On the tenth day, the HET-CAM test was conducted by applying the samples onto the chorioallantoic membrane. The negative control comprised sterile distilled water (H_2_Od), while the positive control was 1% sodium lauryl sulfate (SLS). An amount of 500 mL of each test sample and controls were applied. Observations were made using a Zeiss SteREO Discovery.V8 binocular loupe (Oberkochen, Germany) for 5 min to detect any hemorrhage (blood vessel bleeding), lysis (disintegration of blood vessels), or clotting on the blood vessels (intra or extra-vascular protein denaturalizing). An irritability score (IS) was then calculated based on the recorded occurrences using the following formula:

(1) $$IS\;=\;5\times \left(\frac{301-t_{H}}{300}\right)\;+\;7\times \left(\frac{301-t_{L}}{300}\right)\;+\;9\times \left(\frac{301-t_{C}}{300}\right)$$

where: *H* = hemorrhage; *L* = lysis; *C* = coagulation; *t* = time onset of hemorrhage, lysis, and coagulation reactions on CAM (in seconds) Means values are obtained.

After the calculation of the irritation score (IS), the tested samples can be categorized into distinct groups based on the severity of their irritant properties. These classifications encompass non-irritant samples, falling within the range of 0 to 0.9 on the irritation scale, weak irritants ranging from 1 to 4.9, moderately irritant samples scoring between 5 and 8.9, and lastly, strongly irritant substances with scores ranging from 9 to 21, according to Luepke [130].

#### 4.3.6. Skin Biophysical Parameters Assessment

The animals were divided into four groups (n = 3/group) and were labeled as the following: group 1—mice that received topically the Blank-CP940 hydrogel; group 2—mice that received topically the CAF-CP940 hydrogel; group 3—mice that received topically the CAF-S1-CP940 hydrogel, and group 3—mice that received topically the CAF-S2-CP940 hydrogel. The hydrogels prepared were subjected to a pharmaco-toxicological assessment on sensitive hairless mice. Reference values of skin parameters were collected on the first day before any other activity and the mice were labeled to report any data for a specific individual; all the other data were recorded as differences between the measured values and these references. The same operator to ensure data reliability and accuracy performed the evaluations [150]. The mice were treated with 50 μL of the respective hydrogel on their dorsal skin (the posterior thorax region) every third day and the measurements of various parameters were taken at 20 min post-application. The duration of the experiment was 15 days. Erythema level changes were assessed using a Mexameter^®^ MX 18 probe, the trans-epidermal water-loss (TEWA) with a Tewameter^®^ TM 300 probe, and the hydration of stratum corneum was evaluated with a Corneometer^®^ CM 825 probe. All three probes were connected to a Multiprobe Adapter System (MPA5) from Courage-Khazaka Electronic GmbH (Cologne, Germany), and the readings were obtained through a gentle push on the skin with the specific probes [151,152].

Other parameters were also evaluated, such as the mice’s body weight, behavioral patterns (salivation, tremors, lethargy, sleep, and coma), as well as the appearance of skin modification (after evaluation and measuring the physiological skin parameters values).

#### 4.3.7. Statistical Analysis

For the in vitro experiments, the results were expressed as mean ± standard deviation (SD). To evaluate the statistical differences between carbomer-based hydrogel formulations and control (* *p* < 0.05; ** *p* < 0.01; *** *p* < 0.001; **** *p* < 0.0001), the GraphPad Prism version 9.3.1 (GraphPad Software, San Diego, CA, USA) was used, applying the one-way ANOVA test followed by Dunnet’s multiple comparison post hoc test.

For skin biophysical parameters assessment, the results were expressed as the mean of three independent measurements on the mice’s dorsal skin (n = 3) ± SD. To evaluate the statistical differences between carbomer-based hydrogel formulations containing caffeine and phosphorus derivatives (CAF-CP940; CAF-S1-CP940; CAF-S2-CP940) and Blank-CP940 at *p* ≤ 0.05, the one-way ANOVA test followed by Tukey’s multiple comparison post hoc was applied, using the same version of software [128].

## Figures and Tables

**Table 1:** The carbomer-based hydrogels pH and viscosity values.

Hydrogels Annotation	pH	Apparent Viscosity (Pa.s)	Spreadability (mm^2^)
Blank-CP940	3.96 ± 0.029	0.046 ± 0.16	4654.27 ± 3.091
CAF-CP940	4.83 ± 0.037	0.065 ± 0.34	5671.63 ± 3.898
CAF-S1-CP940	3.32 ± 0.048	0.597 ± 0.52	3846.50 ± 5.477
CAF-S2-CP940	3.71 ± 0.052	0.573 ± 0.81	3957.19 ± 7.001

**Table 2:** The effect of carbomer-based hydrogel formulations containing caffeine and phosphorus derivatives on apoptosis-related gene expression levels between control and (CAF-S1-CP940 and CAF-S2-CP940) in HaCaT cells.

Gene	CAF-S1-CP940	*p*-Value	CAF-S2-CP940	*p*-Value
*Bcl-XL*	−0.241	0.818	1.749	0.134
*BAD*	−0.255	0.806	1.505	0.185
*Caspase-3*	−0.065	0.95	2.518	0.048
*Bcl-2*	−0.866	0.428	1.748	0.134
*Caspase-8*	−0.587	0.586	1.744	0.135
*Bax*	−0.157	0.878	2.927	0.03

**Table 3:** Comparative analyses of the irritation scores (IS) for purified distilled water (H_2_Od), sodium lauryl sulfate 1% (SLS), and carbomer-based hydrogel formulations (CAF-S1-CP940, and CAF-S2-CP940—100 μg/mL), and the occurrence time of hemorrhage, lysis, and coagulation.

Sample/Concentration	Time [seconds]			Irritation Score (IS)
	Hemorrhage (t_H_)	Lysis (t_L_)	Coagulation (t_C_)	
H_2_Od	300	300	300	0.070
SLS 1%	14	50	20	18.070
CAF-S1-CP940 100 μg/mL	300	300	300	0.070
CAF-S2-CP940 100 μg/mL	300	290	300	0.304

**Table 4:** The primer pairs used in the current study.

Genes	Forward	Reverse
*18S*	5′ GTACCCGTTGAACCCCATT 3′	5′CCATCCAATCGGTAGTAGCG3′
*BAD*	5′ CCCAGAGTTTGAGCCGAGTG 3′	5′CCCATCCCTTCGTCCT3′
*Caspase 3*	5′ GCGGTTGTAGAAGAGTTTCGTG 3′	5′CTCACGGCCTGGGATTTCAA 3′
*Caspase 8*	5′ AGAGTCTGTGCCCAAATCAAC 3′	5′GCTGCTTCTCTCTTTGCTGAA 3′
*BCL-XL*	5′ GATCCCCATGGCAGCAGTAAAGCAAG 3′	5′CCCCATCCCGGAAGAGTTCATTCACT 3′
*Bax*	5′ GCCGGGTTGTCGCCCTTTT 3′	5′CCGCTCCCGGAGGAAGTCCA 3′
*Bcl-2*	5′-CGGGAGATGTCGCCCCTGGT-3′	5′-GCATGCTGGGGCCGTACAGT-3′

## Data Availability

The original contributions presented in the study are included in the article, further inquiries can be directed to the corresponding author.
